# The endonuclease activity of MCPIP1 controls the neoplastic transformation of epithelial cells via the c-Met/CD44 axis

**DOI:** 10.1186/s12964-025-02029-x

**Published:** 2025-01-15

**Authors:** Paulina Marona, Rafał Myrczek, Iga Piasecka, Judyta Gorka, Oliwia Kwapisz, Ewelina Pospiech, Janusz Rys, Jolanta Jura, Katarzyna Miekus

**Affiliations:** 1 Department of General Biochemistry, Faculty of Biochemistry, Biophysics and Biotechnology, Jagiellonian University, Krakow, Poland; 2 Doctoral School of Exact and Natural Sciences, Jagiellonian University, Lojasiewicza 11, Kraków, 30-348 Poland; 3 Human Genome Variation Research Group, Malopolska Centre of Biotechnology, Jagiellonian University, Krakow, Poland; 4 Department of Genomics and Forensic Genetics, Pomeranian Medical University in Szczecin, Szczecin, Poland; 5 Department of Tumor Pathology, Centre of Oncology, Maria Skłodowska-Curie Memorial Institute, Cracow Branch, Garncarska 11, Krakow, 31-115 Poland

**Keywords:** MCPIP1, Tumor initiation, Kidney, Neoplastic transformation, CD44, C-Met

## Abstract

The RNase activity of MCPIP1 is essential for regulating cellular homeostasis, proliferation, and tumorigenesis. Our study elucidates the effects of downregulation of MCPIP1 expression and an RNase-inactivating mutation (D141N) on normal epithelial kidney cells, indicating that MCPIP1 expression is a key factor that suppresses neoplastic transformation. We observed that either expression downregulation or mutation of MCPIP1 significantly increased its clonogenicity and altered the expression of cancer stem cell (CSC) markers and factors involved in epithelial-to-mesenchymal transition (EMT). In vivo studies demonstrated that MCPIP1 inactivation in normal epithelial cells leads to significant tumor formation and increased c-Myc phosphorylation, indicating enhanced cell proliferation. Proteomic analysis of mouse plasma revealed increased secretion of cancer-related proteins (CXCL13, CXCL16, and MMP2) in the MCPIP1-mutant group. Additionally, we revealed that MCPIP1 RNase activity regulates the expression of the stemness markers CD44 and CD133 and the phosphorylation of the c-Met receptor in tumor tissue samples. Mechanistically, via coimmunoprecipitation analysis, we found that the RNase activity of MCPIP1 controls CD44 expression and, consequently, that a strong interaction between CD44 and c-Met leads to c-Met activation. This regulation was confirmed in patient samples, in which increased CD44 expression correlated with ccRCC progression. These findings highlight the critical role of MCPIP1 RNase activity in modulating the c-Met/CD44 axis, thereby influencing stemness and tumorigenesis.

**Supplementary Information:**

The online version contains supplementary material available at 10.1186/s12964-025-02029-x.

## Introduction

Tumor-related inflammation, recognized as the seventh hallmark of cancer, plays a crucial role in the earliest stages of neoplastic progression, promoting the development from neoplasia to full-blown cancer. Inflammation contributes to genomic instability, induces epigenetic modifications, and triggers the epithelial-to-mesenchymal transition (EMT), thereby facilitating invasion and metastasis [1–3]. This inflammatory environment supports a multistep cancer initiation process, in which gradual genetic changes convert normal cells into malignant cells. The key players in this transformation include three categories of genes: proto-oncogenes, tumor suppressor genes, and genes related to DNA repair. Mutations in these genes lead to the clonal expansion of cells with the ability to proliferate and self-renew, maintaining an undifferentiated phenotype and expressing specific markers such as CD44 and the c-Met receptor, the protein product of the MET proto-oncogene [4]. CD44 is a nonkinase glycoprotein and a receptor for hyaluronic acid that has upregulated expression in many types of cancer-initiating cells (CICs) or cancer stem cells (CSCs), as well as in rapidly proliferating cells [5]. CD44 expression is associated with EMT and the switch from non-CSCs to CSCs [6, 7]. CD44 is also a coreceptor for certain growth factor receptors, including c-Met, and participates in NF-κB and STAT3 activation, potentiating c-Met signaling [8].

Monocyte chemoattractant protein-1-induced protein 1 (MCPIP1) has been shown to be a potential tumor suppressor that plays an important protective role during renal cell carcinoma progression [9, 10]. Owing to its PilT N-terminus-like (PIN) domain, MCPIP1 expression exerts regulatory control over proinflammatory cytokines, including interleukin-6 (IL-6), tumor necrosis factor-alpha (TNF-α), and interleukin-1β (IL-1β) [11–13]. In addition to its direct impact on inflammation, MCPIP1 exerts regulatory control over pathways associated with cellular proliferation and survival, including the nuclear factor-kappa B (NF-κB) and mitogen-activated protein kinase (MAPK) pathways [14, 15]. The downstream effects of MCPIP1-mediated regulation of these pathways can significantly impact cell cycle progression and apoptosis, which are critical determinants of the early stages of tumorigenesis [16–18]. Additionally, MCPIP1 regulates the expression of the tumor suppressors PTEN, RECK and TIMP3 in cell lines and in in vivo models of ccRCC, indicating the importance of MCPIP1 expression in tumor development and progression [10]. These results underscore the broad genomic protective role of MCPIP1 in the context of tumor initiation. However, the relevant biological functions of MCPIP1 in the early stages of tumor development and its mechanism of action have not been clarified.

In this study, we demonstrated that the RNase activity of MCPIP1 plays a crucial role in governing the initial events leading to neoplastic transformation in normal cells. We found that a lack of MCPIP1 RNase activity induces the expression of cancer stem cell markers such as c-Myc and CD44. This deficiency also leads to the activation of Src kinase and c-MET receptor tyrosine kinase, which influences the maintenance of an undifferentiated/mesenchymal phenotype and initiates tumor development in vivo. Moreover, through its PIN domain, MCPIP1 regulates the expression level of CD44, a coreceptor of c-Met, and consequently induces c-Met receptor phosphorylation. Our findings provide compelling evidence that the absence of MCPIP1 RNase activity in normal epithelial cells modulates factors pivotal in the early stages of tumorigenesis. This deficiency not only influences the microenvironment but is also sufficient to initiate the neoplastic process.

## Results

### A lack of RNase activity or downregulation of MCPIP1 expression enhances clonogenicity and alters the transcriptome

**Fig. 1:**
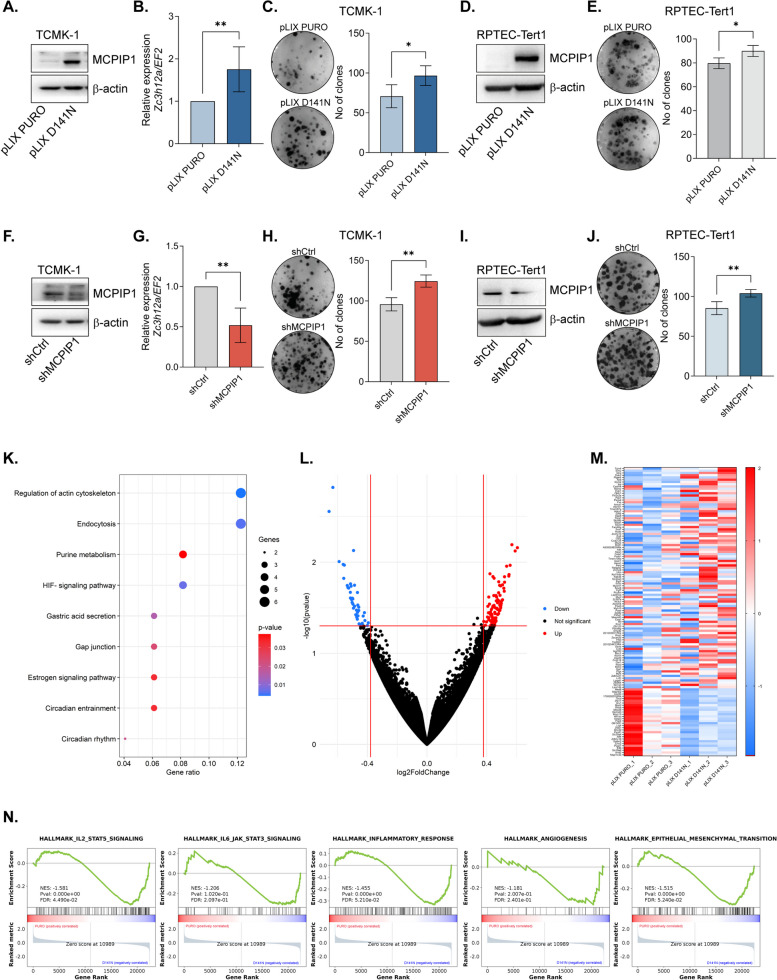
MCPIP1 effect on clonogenicity **A**. Representative western blot for MCPIP1 in TCMK-1 control cells (pLIX PURO) and cells with MCPIP1 mutation (pLIX D141N) with β-actin as a loading control. **B** Expression of *Zc3h12a* in TCMK-1 cells. **C** Representative images of clones formed by TCMK-1 control cells (pLIX PURO) and cells with MCPIP1 mutation (pLIX D141N) with quantification of clones number. **D** Representative western blot for MCPIP1 in RPTEC-Tert1 control cells (pLIX PURO) and cells with MCPIP1 mutation (pLIX D141N) with β-actin as a loading control. **E** Representative images of clones formed by RPTEC-Tert1 control cells (pLIX PURO) and cells with MCPIP1 mutation (pLIX D141N) with quantification of clones number. **F** Representative western blot for MCPIP1 in control TCMK-1 cells (shCtrl) or cells with MCPIP1 downregulation (shMCPIP1) with β-actin as a loading control. **G** Expression of *Zc3h12a* in TCMK-1 cells. **H** Representative images of clones formed by TCMK-1 control cells (shCtrl) and cells with MCPIP1 downregulation (shMCPIP1) with quantification of clones number. **I** Representative western blot for MCPIP1 in RPTEC-Tert1 control cells (shCtrl) and cells with MCPIP1 downregulation (shMCPIP1) with β-actin as a loading control. or MCPIP1 downregulation (shMCPIP1) **J** Representative images of clones formed by RPTEC-Tert1 control cells (shCtrl) and cells with MCPIP1 downregulation (shMCPIP1) with quantification of clones number. The results are presented as the mean ± SD of at least three independent experiments. *P* values were estimated using Student *t*-test. *P < 0.05; **P < 0.01. **K** Gene onthology (GO) enrichment analysis of upregulated biological processes in TCMK-1 pLIX D141N cells compared to control cells (TCMK-1 pLIX PURO). **L** Volcano plot representing differentially expressed genes (DEGs) in TCMK-1 pLIX D141N cells compared to control cells. **M** Heatmap illustrating the expression levels of most changed genes. **N** Enrichment plots from gene set enrichment analysis (GSEA) TCMK-1 pLIX D141N vs TCMK-1 pLIX PURO

In our recent findings, we demonstrated that both the downregulation of expression and the mutation (D141N) of MCPIP1 lead to the complete elimination of its RNase function, resulting in increased proliferation of the kidney cancer cell lines Caki-1 and Caki-2 [9]. Expanding our investigation to normal epithelial kidney cells from mice (TCMK-1 cells) and normal human epithelial renal cell line (RPTEC-Tert1), we observed a notable increase in clone formation upon mutation of MCPIP1 (pLIX D141N) (Fig. 1A-E). Similarly, the downregulation of MCPIP1 expression increased the number of clones of both TCMK-1 and RPTEC-Tert1 cells (Fig. 1F-J).

Subsequent next-generation sequencing of TCMK-1 control cells (pLIX PURO) and cells with an MCPIP1 mutation (pLIX D141N) revealed alterations in signaling pathways such as the regulation of the actin cytoskeleton, endocytosis, and the HIF signaling pathway, along with changes in gap junctions (Fig. 1K). Further analysis revealed genes with down- and upregulated expression, and the validation of selected genes confirmed significant changes in the expression of *Mdm2*, *Fgf7*, *P4ha1*, and *Hif1an* (Fig. 1L, M; Supplementary Fig. 1). Notably, the expression of *Mdm2*, a potent tumor oncogene, significantly increased in tissues with MCPIP1 mutations. Additionally, the expression of *Fgf7* and *P4ha1* was elevated, whereas the expression of the Hif1 inhibitor *Hif1an* was decreased in the pLIX D141N group (Supplementary Fig. 1). In addition, we performed gene set enrichment analysis and found significant enrichment of activity in the IL-2/STAT5 pathway, the EMT pathway, the IL-6/JAK/STAT3 pathway, the inflammatory response pathway and the angiogenesis pathway in TCMK-1 pLIX D141N cells (Fig. 1N).

### MCPIP1 expression levels and activity regulate key factors involved in the EMT, proliferation and stemness

**Fig. 2:**
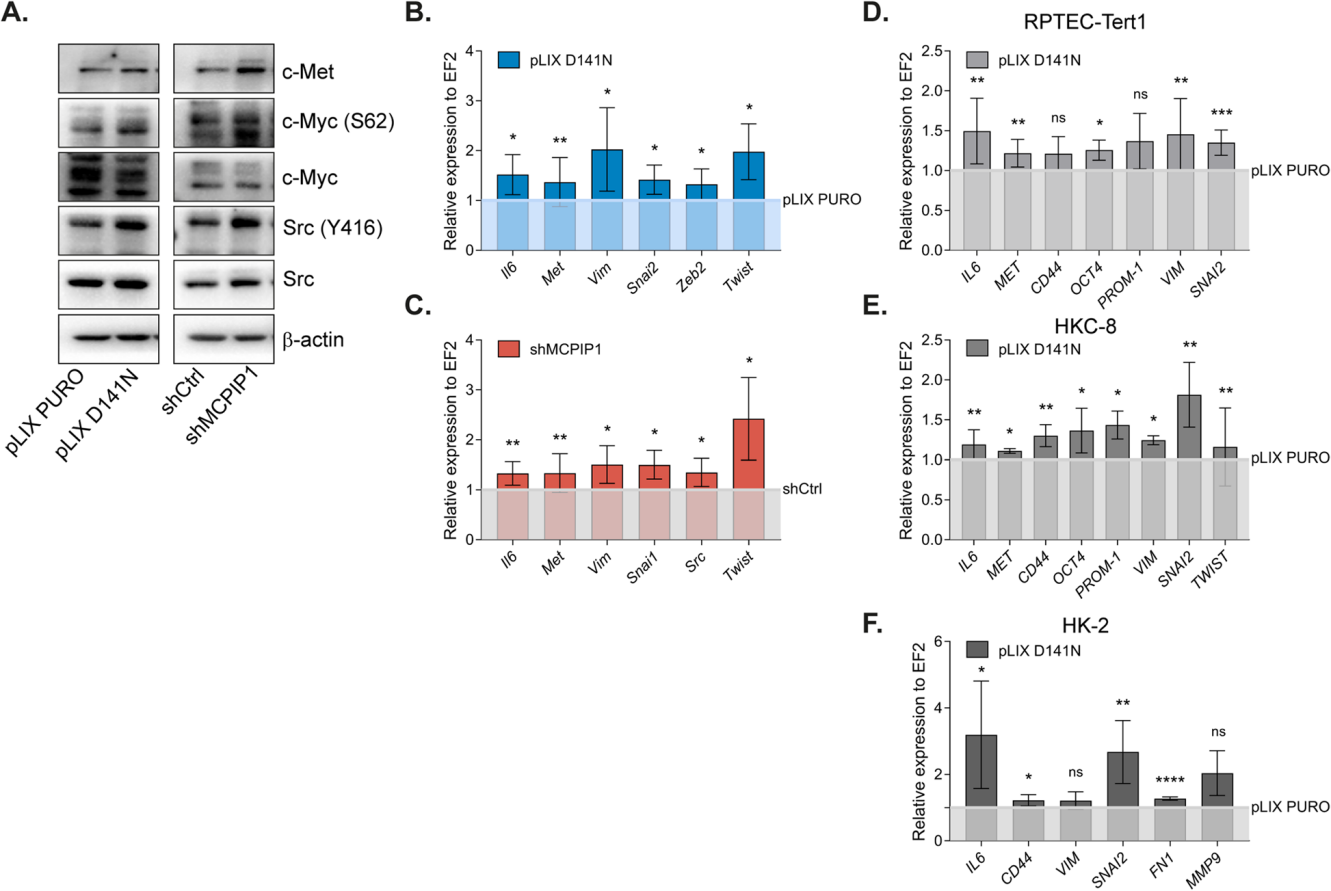
Level of stemness, proliferation and EMT markers in various cell lines with different MCPIP1 activity. **A** Representative western blot of TCMK-1 control cells (pLIX PURO or shCtrl) and cells with mutation (pLIX D141N) or cells with MCPIP1 downregulation (shMCPIP1) with β-actin as a loading control. **B** Expression of *Il6, Met, Vimentin, Snai2, Zeb2* and *Twist* in TCMK-1 cells. The light blue line represent control sample. *P* values were estimated using Student *t*-test (*Il6, Vimentin*) or Mann–Whitney test (*Met, Snai2, Zeb2, Twist*). **C** Expression of *IL-6, Met, Vimentin, Snai1, Zeb2* and *Twist* in TCMK-1 cells. The light grey line represent control sample. *P* values were estimated using Student *t*-test (*Il6, Src*) or Mann–Whitney test (*Met, Vimentin, Twist, Snai1*). **D** Expression of *IL6, MET, VIMENTIN, CD44, PROM1, OCT4, SNAI2* in RPTEC-Tert1 cell line control (light grey line) vs pLIX D141N. **E** Expression of *IL6, MET, VIMENTIN, CD44, PROM1, OCT4, SNAI2* in HKC-8 cell line pLIX PURO (light grey line) vs pLIX D141N. **F** Expression of *IL6, CD44, VIM, SNAI2, FN1, MMP9* in HK-2 cell line pLIX PURO (light grey line) vs pLIX D141N. *P* values were estimated using Student *t*-test (*CD44, PROM1, SNAI2*) or Mann–Whitney test (*MET, IL6, VIM, OCT4*). The results are presented as the mean ± SD of at least three independent experiments. *P < 0.05; **P < 0.01; ***P < 0.001; ****P < 0.0001

Our results indicate that the lack of MCPIP1 endonuclease activity in TCMK-1 cells resulted in increased levels of proteins including c-Myc and the signaling molecules Src kinase and the c-Met receptor (Fig. 2A). Furthermore, our observations revealed an upregulation of expression of factors associated with EMT, such as *Vimentin* and the transcription factors *Snai1, Zeb2*, and *Twist* (Fig. 2B). Similarly, MCPIP1 expression downregulation resulted in increased expression levels of *c-Myc, Src,* and EMT markers *Vimentin, Snai1* and *Twist* (Fig. 2C). Both expression downregulation and mutation of the PIN domain led to increased expression of *Il6*, a well-known factor involved in inflammation, tumorigenesis, and angiogenesis and a direct target of MCPIP1 activity (Fig. 2B, C). Because we observed that a lack of endonuclease activity in normal murine cells changed the expression of key factors involved in tumorigenesis, inflammation and EMT, we speculated whether we would observe similar changes in human cell lines. To verify our hypothesis, we transduced normal renal epithelial cell lines RPTEC-Tert1, HKC-8 and HK-2 to overexpress a mutated form of MCPIP1 lacking RNase activity. We found that mutation of MCPIP1 induces increased expression of genes associated with stemness and neoplastic transformation in human epithelial cells. We observed increased expression of *IL6*, *MET* and *VIMENTIN*, as well as increased expression of factors involved in the acquisition of stemness features such as *CD44*, *PROM1* and *OCT4* (Fig. 2D-F). These results suggest that MCPIP1 activity is extremely important for maintaining cell homeostasis in both murine and human normal cells.

### A lack of MCPIP1 RNase activity leads to tumor formation in vivo

**Fig. 3:**
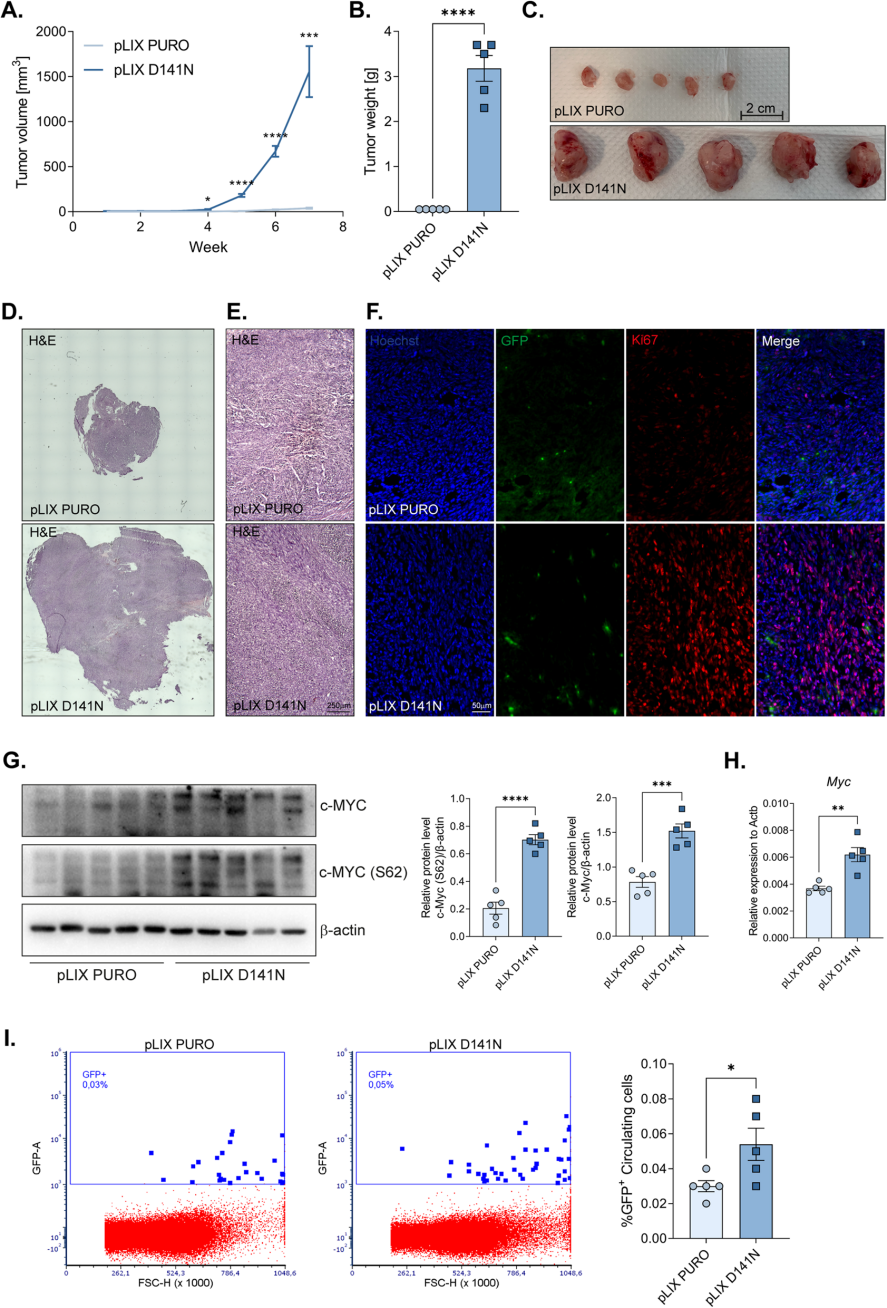
Effect of MCPIP1 mutation on tumor growth in vivo. **A** Caliper measurements of tumor volume during 7 weeks. **B** Weight of tumors. **C** Representative images of extracted tumors. **D**, **E** Representative images of tumor sections after hematoxylin and eosin staining. **F** Ki67 immunofluorescent staining of OCT tumor sections, representative images with Hoechst used to visualize nuclei. TCMK-1 cells were GFP positive. **G** Western blot result of tumors fragments with β-actin as a loading control. On the right densitometric analysis. **H** Expression of *Myc* in tumor samples. **I** Flow cytometer analysis of circulating GFP-positive TCMK-1 cells in mouse lysed blood. Tumors and blood were collected 7 weeks after subcutaneous injection of cells. Animal studies involved 10 NOD-SCID mice: TCMK-1 pLIX PURO, *N* = 5; TCMK-1 pLIX D141N, *N* = 5. The results are presented as the mean ± SD. *P* values were estimated using Student *t*-test. *P < 0.05; **P < 0.01; ***P < 0.001; ****P < 0.0001

Our recent findings revealed that MCPIP1 expression downregulation or mutation results in increased tumor formation and metastasis [9]. While TCMK-1 cells are not inherently tumorigenic after injection into immunosuppressed mice, we sought to investigate whether a lack of MCPIP1 activity could induce tumor growth in vivo. Upon subcutaneous injection of 5 million GFP-positive cells into NOD-SCID mice, tumor-like structures emerged within seven weeks (Fig. 3A-C). Notably, compared with control cells, MCPIP1-mutant cells formed substantial tumors, whereas minimal structures formed in the control group (Fig. 3A-C). Similar, although statistically insignificant, results were observed following downregulation of MCPIP1 expression (Supplementary Fig. 2 A-C). Histological examination through hematoxylin/eosin staining revealed strikingly similar tumor structures between the groups (Fig. 3D, E; Supplementary Fig. 2 D). However, we observed a significant disparity in the number of GFP-positive cells within the tumor mass and an increased signal of Ki67 expression in the pLIX D141N group compared with the controls (Fig. 3F). The differences in tumor volume, weight, and Ki67 expression levels may be explained by increased phosphorylation of the key proliferation marker c-Myc in pLIX D141N tumors (Fig. 3G, H). In the MCPIP1 expression downregulation group, we did not observe changes in Ki67 expression levels, but there was a significant increase in c-Myc phosphorylation compared with that in the control group (Supplementary Fig. 2 E–G).

Additionally, analysis of mouse blood revealed an increased presence of circulating GFP-positive cells in the group with the MCPIP1 mutation compared with the control group (Fig. 3I). Similar trends, although not statistically significant, were noted after downregulation of MCPIP1 expression (Supplementary Fig. 2 H, Supplementary Fig. 3).

### MCPIP1 RNase activity influences alterations in secreted cancer-related proteins and local fibrosis

**Fig. 4:**
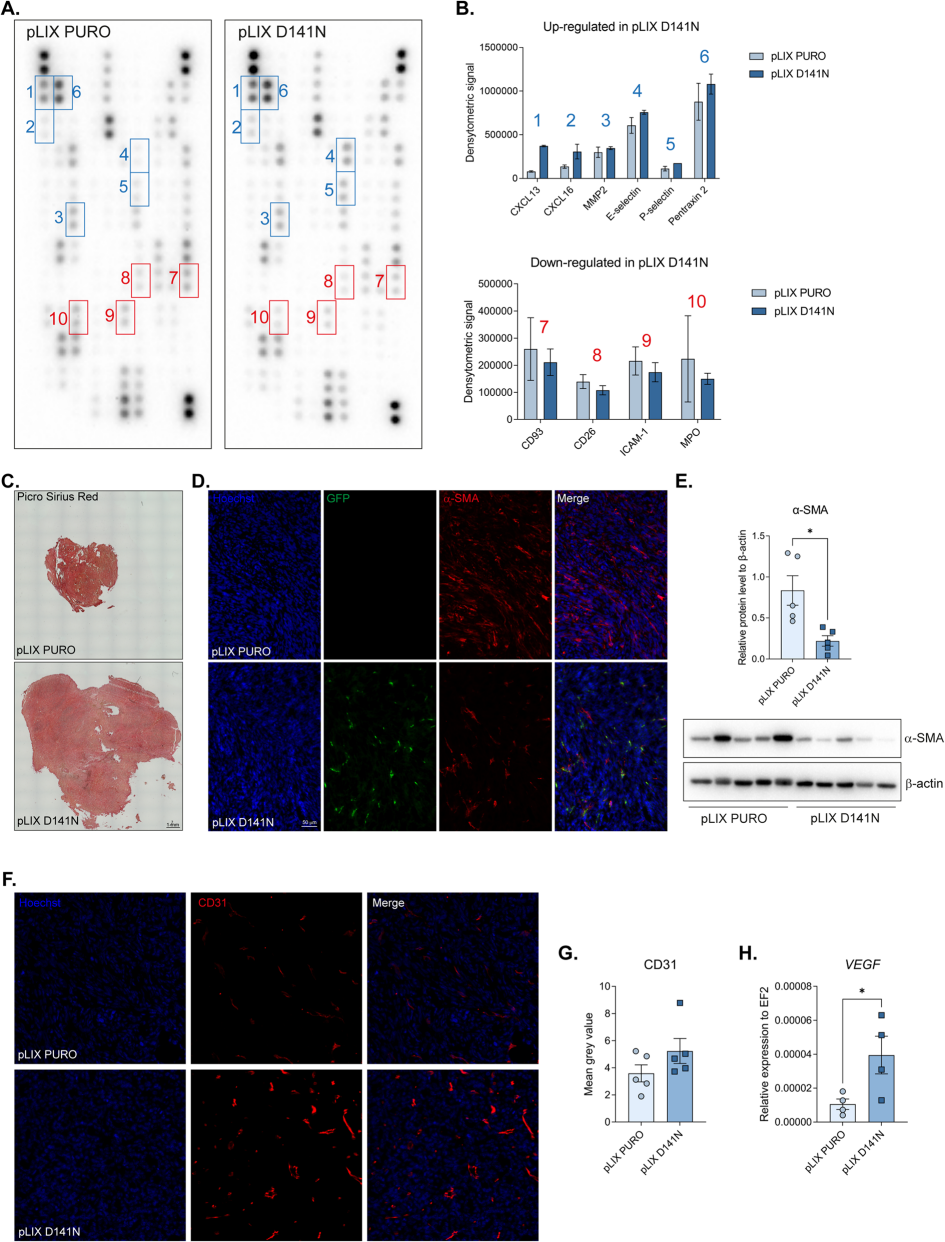
Effect of MCPIP1 mutation on local microenvironment. **A** Representative images of Proteome Profiler membranes. Up-regulated targets in pLIX D141N are marked with blue, whereas downregulated are marked with red. **B** Proteome Profiler densitometric analysis from 4 mice serum samples (*N* = 2 per group). **C** Representative images of tumor sections stained with Picro Sirius Red. **D** α-SMA immunofluorescent staining of OCT tumor sections, representative images with Hoechst used to visualize nuclei. TCMK-1 cells were GFP positive. **E** Densitometric analysis and western blot from tumors fragments with β-actin as a loading control. **F** CD31 immunofluorescent staining of OCT tumor sections, representative images with Hoechst used to visualize nuclei. **G** Quantification of fluorescence intensity presented as mean grey value. **H** Expression of *Vegf* in tumor sections. Animal studies involved 10 NOD-SCID mice: TCMK-1 pLIX PURO, *N* = 5; TCMK-1 pLIX D141N, *N* = 5, except *Vegf* expression where *N* = 4 for each group. The results are presented as the mean ± SD. *P* values were estimated using Student *t*-test. *P < 0.05

To assess the impact of the MCPIP1 expression level and activity on the local microenvironment, we conducted proteomic profiling. Analysis of mouse plasma revealed increased secretion of cancer-related proteins, such as CXCL13, CXCL16, MMP2, selectins, and pentraxin 2, in the group with MCPIP1 mutations. Interestingly, the control group exhibited increased secretion of factors associated with local fibrosis and inflammation, including CD93, CD26, ICAM-1, and MPO (Fig. 4A, B; Supplementary Fig. 4). Considering the observed changes in tumor growth and protein secretion into the plasma, we stained tumor sections with Picro Sirius Red, which revealed collagen fibers throughout the entire tumor area (Fig. 4C). α-SMA staining and Western blot analysis revealed that control tumors contained more fibroblasts expressing α-SMA than pLIX D141N tumors did, suggesting that the structures observed were indicative of local fibrosis induced by the injection of TCMK-1 cells rather than tumor formation (Fig. 4D, E). Notably, we did not observe significant changes in the expression levels of α-SMA or Picro Sirius Red staining in MCPIP1-downregulated tumors compared with control tumors (Supplementary Fig. 5A, B).

Staining for CD31 (platelet endothelial cell adhesion molecule, PECAM-1), a vascular marker, showed an increase in the number of CD31 positive cells in tumors formed by MCPIP1-mutated cells (Fig. 4F, G). Further analysis of tumors revealed that the lack of RNase activity of MCPIP1 led to an increase expression of proangiogenic vascular endothelial growth factor (VEGF) which promotes the recruitment of endothelial precursor cells (Fig. 4H).

### MCPIP1 expression orchestrates the influx of immune cells into tumors

**Fig. 5:**
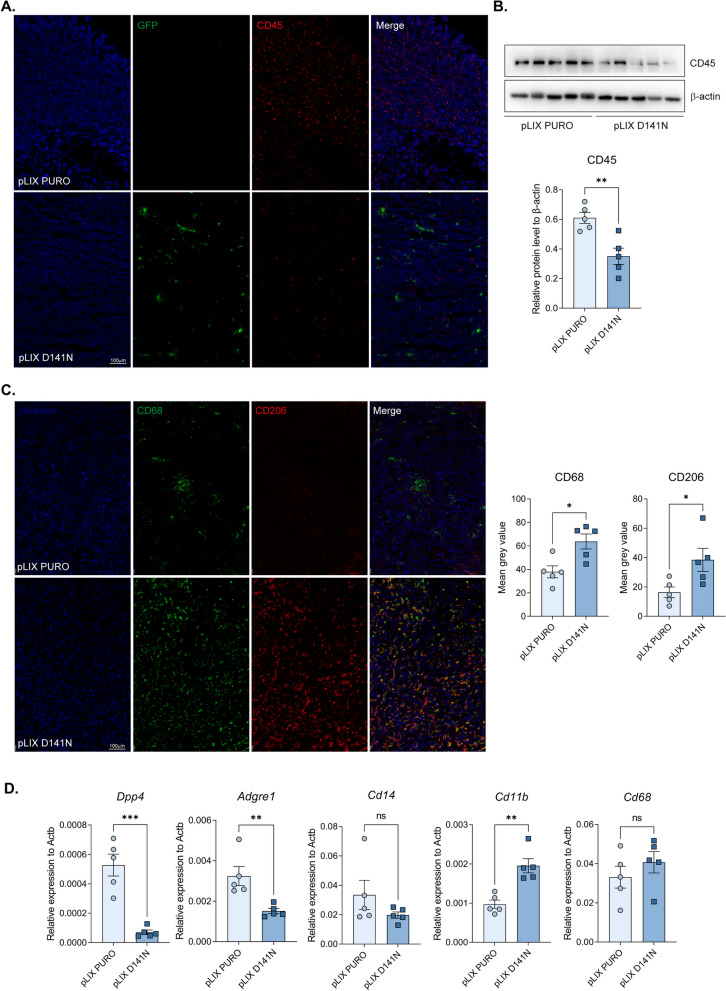
Effect of MCPIP1 mutation on local inflammation. **A** CD45 immunofluorescent staining of OCT tumor sections, representative images with Hoechst used to visualize nuclei. TCMK-1 cells were GFP positive. **B** Western blot result of tumors fragments with β-actin as a loading control. On the right densitometric analysis. **C** CD68 and CD206 immunofluorescent staining of OCT tumors sections, representative images with Hoechst used to visualize nuclei. On the right quantification of fluorescence intensity presented as mean grey value. **D** Relative expression of immune response factors such as *Dpp4*, *Adgre1*, *Cd14*, *Cd11b* and *Cd68* in tumor tissue.. Animal studies involved 10 NOD-SCID mice: TCMK-1 pLIX PURO, *N* = 5; TCMK-1 pLIX D141N, *N* = 5. The results are presented as the mean ± SD. *P* values were estimated using Student *t*-test (CD45, *Dpp4*, *Cd11b*, *Cd68*) or Mann–Whitney (*Cd206*, *Adgre1*, *Cd14*). *P < 0.05; **P < 0.01; ***P < 0.001

To further examine the influence of MCPIP1 expression on the local microenvironment, we analyzed the influx of immune cells. CD45 staining and Western blot analysis revealed a greater presence of CD45-positive cells in control tumors than in those containing the pLIX D141N mutation (Fig. 5A, B). Conversely, tumors expressing inactive MCPIP1 displayed increased infiltration of both CD68 and CD206 positive cells and more expression of *Cd11b* and *Cd68* (Fig. 5C, D) which may suggest more M2 macrophages in these tumors. Additionally, control tumors presented increased expression of *Dpp4*, *Adgre1* (encoding F4/80), indicating an influx of myeloid-lineage cells (Fig. 5C). These findings suggest that while control tumors had a lower proliferation rate, they experienced an influx of immune cells, including macrophages and fibroblasts, resulting in local fibrosis with enhanced inflammation. Conversely, MCPIP1 mutation led to increased cell proliferation coupled with fibrosis and inflammation, together with increased infiltration of M2 macrophages, resulting in larger tumors with more aggressively proliferating cells (Fig. 5D). Moreover, we observed more CD45-positive cells and increased expression of *Adgre1*, *Cd14*, *Cd11b*, and *Cd68* in tumors with downregulated MCPIP1 expression than in control tumors (Supplementary Fig. 5C-E).

### Tumors expressing MCPIP1 mutations presented elevated expression of factors associated with neoplastic transformation

**Fig. 6:**
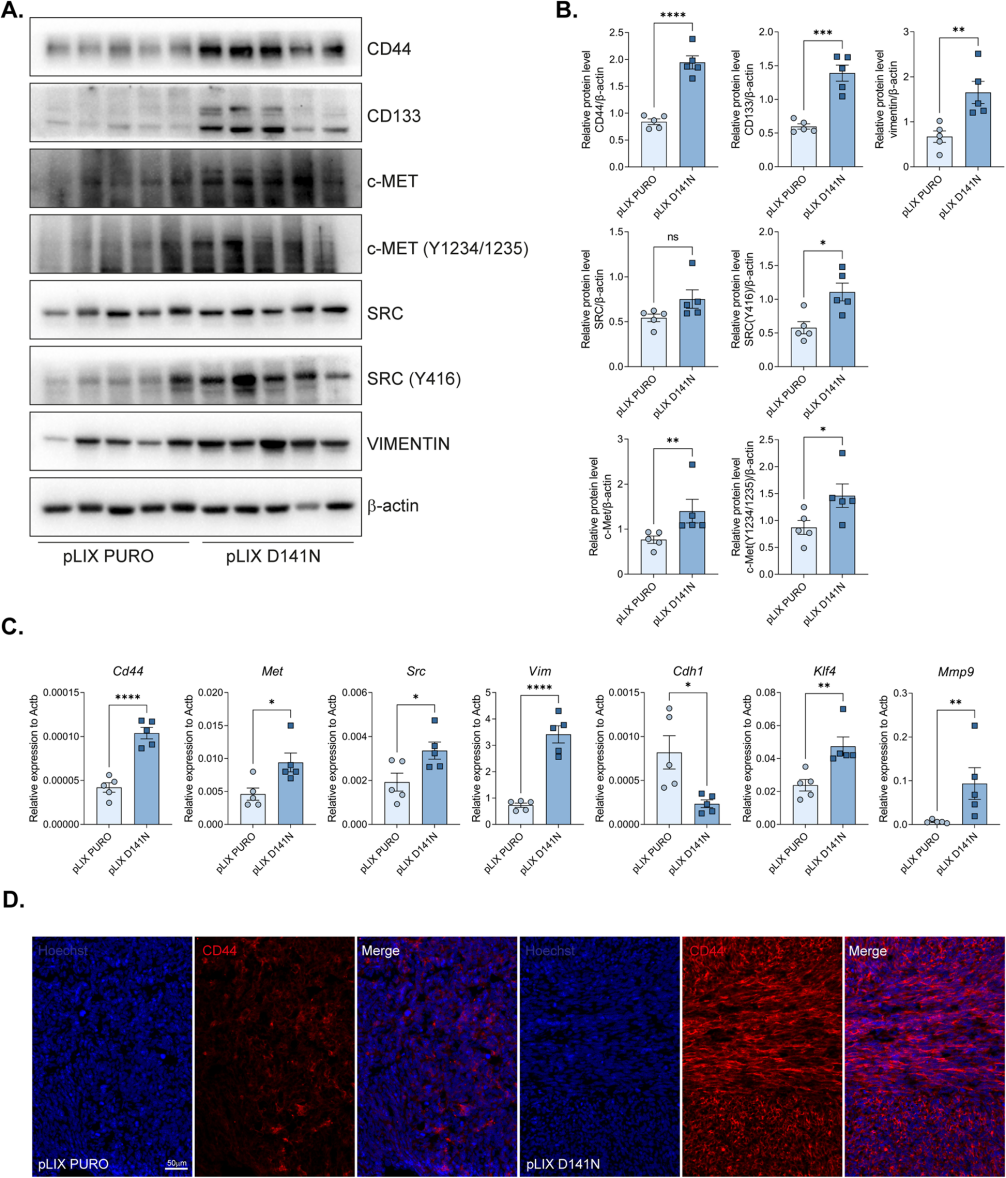
Effect of MCPIP1 mutation on changes in factors involved in tumor cell stemness and EMT. **A** Western blot result of tumors fragments with β-actin as a loading control. **B** Densitometric analysis of western blot results. **C** Relative expression of factors involved in tumor growth, progression and EMT *Cd44*, *Met*, *Src*, *Vim*, *Cdh1*, *Klf4* and *Mmp9* in tumor tissues. **D** CD44 immunofluorescent staining of OCT tumor sections, representative images with Hoechst used to visualize nuclei. Animal studies involved 10 NOD-SCID mice: TCMK-1 pLIX PURO, *N* = 5; TCMK-1 pLIX D141N, *N* = 5. The results are presented as the mean ± SD. *P* values were estimated using Student *t*-test or Mann–Whitney test (MET, SRC, *Klf4*, *Mmp9*). *P < 0.05; **P < 0.01; ***P < 0.001; ****P < 0.0001

We observed increased expression of stemness markers, such as CD44 and CD133, in tumors expressing inactive MCPIP1, along with increased phosphorylation of the c-Met receptor, Src kinase, and vimentin (Fig. 6A, B). Additionally, we noted similar alterations in the expression of *CD44, Klf4, Met, Src, Vimentin*, and *MMP9* (Fig. 6C). Furthermore, there was a significant decrease in the expression of *Cdh1*, a key marker of epithelial cells (Fig. 6C). A similar pattern was found in tumors with downregulated MCPIP1 expression; however, these changes did not reach statistical significance (Supplementary Fig. 6A‒C). pLIX D141N and shMCPIP1 tumors demonstrated an increase in CD44 staining compared with control tumors, indicating that the RNase activity of MCPIP1 regulates CD44 expression in ccRCC cells. These results suggest that MCPIP1 expression influences the stemness potential of cells, leading to neoplastic transformation (Fig. 6D).

### The enzymatic activity of MCPIP1 regulates the c-Met/CD44 axis

**Fig. 7:**
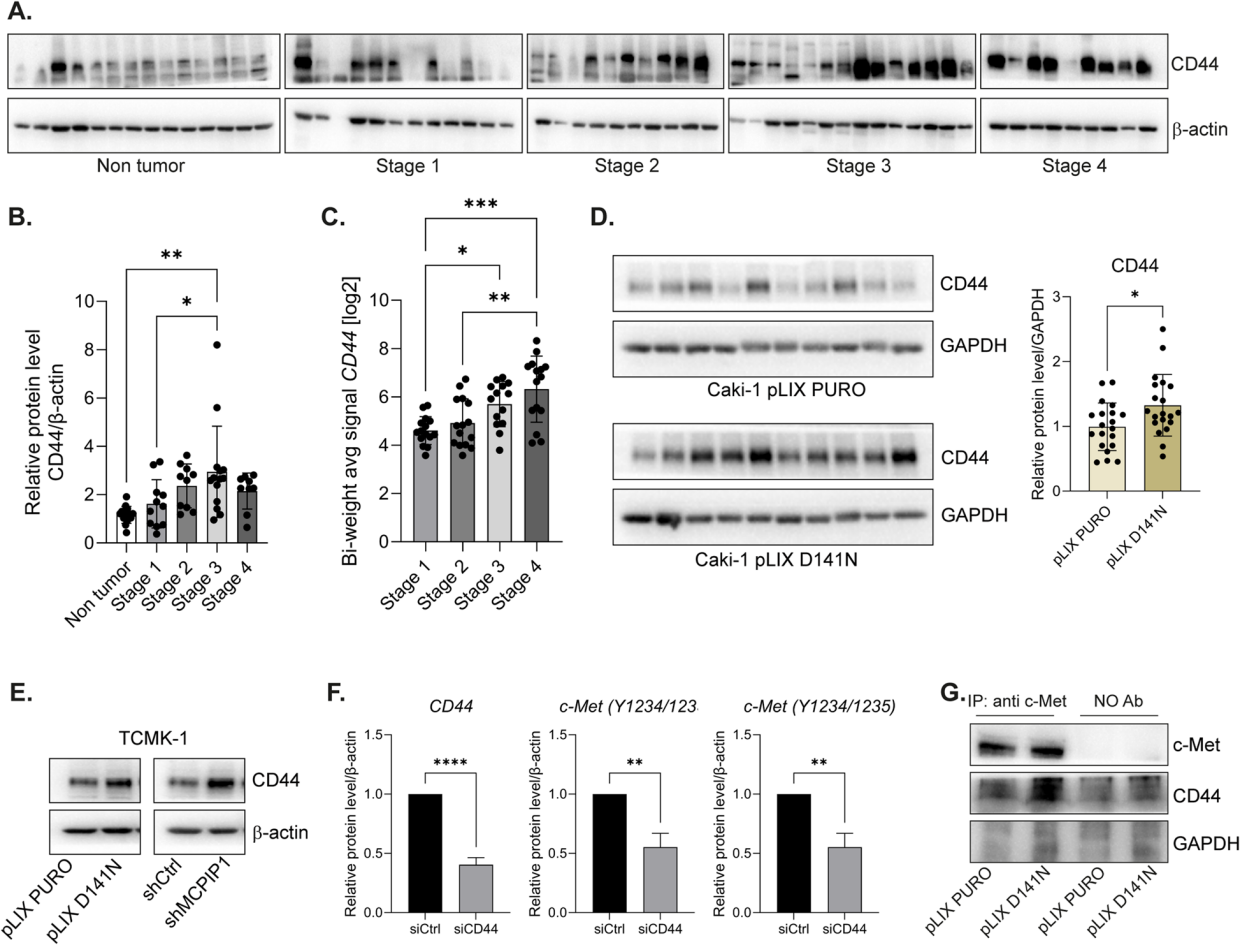
The effect of MCPIP1 on c-Met/CD44 axis. **A** Western blot result of tissue fragments from patients with ccRCC (stage 1 *N* = 11, stage 2 *N* = 10, stage 3 *N* = 14, stage 4 *N* = 9) or adjacent, healthy tissue (non-tumor *N* = 14) with β-actin as a loading control. The results are presented as the mean ± SD. *P* values were estimated using One-way ANOVA. **B** Densitometric analysis of western blot results. **C** Quantification of the signal from microarray. Statistical analysis was performed using one-way ANOVA between subjects (unpaired) where *N* = 15 per group. **D** Western blot from tumor specimens, collected 6 weeks after Caki-1 (pLIX PURO or pLIX D141N) cell injections, with GAPDH as the loading control. *N* = 10 per group. Graph with densitometric quantification of western blot. **E** Representative western blot of CD44 in TCMK-1 cells with MCPIP1 mutation (pLIX D141N) or downregulation (shMCPIP1). **F** Densitometric analysis of western blot results of Caki-1 cells after transfection with control siRNA or siCD44. The results are presented as the mean ± SD of three independent experiments. *P* values were estimated using Student *t*-test *P < 0.05; **P < 0.01; ***P < 0.001; ****P < 0.0001. **G** Representative western blot after co-immunoprecipitation of c-Met and CD44 in Caki-1 (pLIX PURO or pLIX D141N) cells

CD44, a nonkinase transmembrane glycoprotein, is implicated in the progression of several cancers, including ccRCC. CD44 is also a molecular marker for cancer stem cells (CSCs) and has been found to play a role in the EMT process [19, 20]. Analysis of patient samples revealed increased expression of CD44 protein in ccRCC tissues compared with healthy, adjacent tissues. We found that expression of CD44 increased with disease progression (Fig. 7A, B). Similar results were obtained via a gene expression microarray of samples from 60 patients with ccRCC (Fig. 7C). We show that the level of CD44 increased together with ccRCC progression (1–4 gradings) (Fig. 7A-C). Next, we evaluated the expression of CD44 in tumors consisting of ccRCC cell line Caki-1 and detected higher protein expression of CD44 in tumors containing the MCPIP1 mutation (pLIX D141N) than in control tumors (pLIX PURO) (Fig. 7D). Our results show that lack of RNase activity of MCPIP1 significantly increases CD44 levels, suggesting that CD44 is dependent on MCPIP1 activity. (Fig. 7D, E, Supplementary Fig. 7A).

CD44 is known to act as a coreceptor of c-Met. The tyrosine kinase receptor c-Met has been identified as an essential factor responsible for the functional CSC phenotype and for sustaining the undifferentiated/mesenchymal phenotype as well as a significant factor for the initialization of tumor growth under in vivo conditions [21]. Previously, we reported that MCPIP1 expression decreases during ccRCC progression and that low expression of MCPIP1 negatively correlates with the expression of the c-Met receptor [9, 15]. We observed that silencing expression of the CD44 receptor led to a significant decrease in the expression and phosphorylation of the c-Met receptor (Fig. 7F, Supplementary Fig. 7B-D). c-Met recruits co-factors to amplify signaling and facilitate receptor activation. One key inducer of c-Met activation is CD44, which synergizes with c-Met to enhance signal transduction through the CD44/c-Met complex [8]. We detected increased colocalization of CD44 and c-Met expression in cells lacking MCPIP1 RNase activity. Coimmunoprecipitation analysis revealed a strong connection between c-Met and CD44 expression, especially in cells lacking the enzymatic activity of MCPIP1 (Fig. 7G), indicating that the RNase activity of MCPIP1 controls the expression of CD44 and, in turn, the phosphorylation of c-Met.

## Discussion

Owing to its function as a negative regulator of inflammation, the MCPIP1 protein has become an interesting subject of research because of its potential role in the development of cancer [22]. MCPIP1 is a member of the Regnase family and is composed of a PilT N-terminus-like domain that is responsible for the mRNA degradation of major proinflammatory cytokines, such as IL-6, IL-1β, and IL-12b, following physical interactions with stem‒loop structures in the 3′ UTRs of the transcripts [23, 24]. MCPIP1, in addition to regulating the stability of transcripts for typical proinflammatory factors and cytokines, negatively affects factors involved in apoptosis, proliferation and angiogenesis in cancer cells [9, 16].

While they have been extensively studied in the context of immune response modulation and processes involved in cancer biology, the relevant biological functions of MCPIP1 and its mechanism of action influencing the initial stages of tumor development have not been clarified. In this study, we demonstrated for the first time that MCPIP1 activity may influence the phenotypes of normal cells, activate events that lead to neoplastic transformation in normal cells and initiate tumor development in vivo. Our findings provide compelling evidence that the absence of the RNase activity of MCPIP1 in normal epithelial cells modulates factors pivotal in the early stages of tumorigenesis and leads to neoplastic transformation in normal cells.

Initially, in our study, we reported that the complete elimination of the RNase function of MCPIP1 through a single mutation, D141N, resulted in increased clonogenicity in normal epithelial renal cells and alterations in signaling pathways regulating the actin cytoskeleton, endocytosis, and the HIF signaling pathway. Gene set enrichment analysis revealed significant enrichment of the epithelial-to-mesenchymal transition pathway, the IL-6/JAK/STAT3 pathway, the inflammatory response pathway and the angiogenesis pathway. We also detected increased expression of key factors involved in tumorigenesis, including proliferation activators; epithelial-to-mesenchymal transition inducers; Snai1, Snai2, Zeb2, and Twist; the mesenchymal marker vimentin; and stemness factors c-Met, CD44, and Oct4. MCPIP1 expression has been shown to negatively regulate HIF1α and HIF2α expression and influence the expression levels of the tumor suppressors PTEN and RECK in ccRCC cell lines, indicating the importance of MCPIP1 expression in ccRCC development and progression [10, 14]. Our previous results also revealed that the RNase activity of MCPIP1 regulates the expression of proangiogenic IL-8 and VEGF and proinflammatory IL-6 and promotes the phosphorylation of c-Met in ccRCC cells [9]. Moreover, we found that MCPIP1 expression is crucial for the expression of mesenchymal markers and prevents epithelial-to-mesenchymal progression in normal RPTEC/TERT1 cells [25].

Our previous findings revealed that MCPIP1 expression silencing is correlated with increased tumor growth and metastasis in a xenotransplant model of ccRCC. However, its mechanism of action influences the early stages of tumor development, and the influence of MCPIP1 expression on cellular and molecular biological regulation has not been clarified. In this study, we used murine TCMK-1 cells, which are not tumorigenic in immunosuppressed mice. However, after subcutaneous injection, TCMK-1 cells lacking MCPIP1 RNase activity (pLIX D141N) formed large tumors, whereas injection of control cells (pLIX PURO) formed only local fibrosis. Tumors generated from cells lacking MCPIP1 endonuclease activity are characterized by increased expression of Ki67 together with the expression and activation of the key proliferation marker c-Myc. MYC is one of the most potent oncogenes, and MYC activation is necessary for the initiation of tumorigenesis [26]. Stable overexpression of MCPIP1 in the BE [2]-C line resulted in a significant decrease in MYCN expression, proliferation potential and viability [27]. However, MYC activation alone is incapable of inducing the proliferation or neoplastic transformation of most normal human cells and generally cannot induce tumorigenesis [26, 28]. MYC cooperates with many other oncogenic precursors, such as antiapoptotic proteins of the BCL2 family, to initiate tumorigenesis [29]. We previously reported that the RNase activity of MCPIP1 regulates the expression of apoptosis-related transcripts and that a lack of MCPIP1 RNase activity increases *BCL-2* expression in renal carcinoma cells [10]. Moreover, MCPIP1 expression has been shown to act as a potent tumor suppressor in breast cancer cells by inducing tumor apoptosis through selectively suppressing the expression of antiapoptotic gene transcripts, including *Bcl2L1 and Bcl2A1* [16]*.* In this study*,* we show that, owing to its RNase activity*,* MCPIP1 expression regulates one of the important oncogenes in the neoplastic cascade*,* thus activating cell proliferation.

Inflammation, recognized as the seventh hallmark of cancer, is regulated by a variety of factors, including MCPIP1 expression, which plays a critical downregulatory role in the inflammatory response [30]. The endoribonuclease activity of MCPIP1 exerts regulatory control over inflammatory pathways by selectively degrading specific mRNAs implicated in cytokine production [23]. Our findings provide compelling evidence that the absence of MCPIP1 RNase activity in normal epithelial cells modulates factors pivotal in the early stages of tumorigenesis. In addition to its influence on cytokines, MCPIP1 expression plays a crucial role in modulating immune response elements integral to tumor initiation and contributes to shaping the immune landscape within the TME. In our study, we observed increased infiltration of both CD68 and CD206 positive cells suggesting a higher presence of M2-type tumor-associated macrophages (TAMs) in tumors lacking MCPIP1 RNase activity. M2 macrophages are known to release immunosuppressive cytokines, such as transforming growth factor-beta (TGF-β) and VEGF, which contribute to tumor progression, angiogenesis and drug resistance [31]. In kidney cancer, M2-like TAMs promote cancer progression through angiogenesis and stimulation of tumor proliferation [32].

Our findings suggest that the absence of MCPIP1 in premalignant cells alters the inflammatory microenvironment by promoting increased infiltration of M2 macrophages, as well as the release of VEGF and IL-6, which drive angiogenesis and tumor progression Future studies will be necessary to elucidate the role of MCPIP1 activity in shaping the tumor microenvironment during tumor development, and to further explore the mechanisms by which MCPIP1 expression may facilitate escape from adaptive immunity.

Our study revealed increased expression of c-Myc together with high expression of the mesenchymal markers VIM (Vimentin), FN1 (Fibronectin), ZEB2 (SIP-1), FOXC2, SNAIL1 (Snail), SNAIL2 (Slug), and TWIST and the cancer stem-like cell marker CD44 in cells and tumors lacking MCPIP1 RNase activity. It has been postulated that the EMT process promotes the generation of self-renewing stem cells with high expression of mesenchymal markers and CD44 [33]. Gastrointestinal cancer cells can also be induced to express an embryonic stem-like state via the forced expression of Oct3/4, Sox2, Klf4, and c-Myc, and the overexpression of E box-binding transcription factors can induce differentiated somatic cells to generate neoplastic stem cells [33, 34]. We previously demonstrated that MCPIP1 expression regulates EMT and the acquisition of a mesenchymal phenotype by influencing the activity of the β-catenin pathway in renal cells [25]. Our previous findings also revealed increased expression of the c-Met receptor in ccRCC tumor cells lacking MCPIP1 expression [15]. c-Met mRNA and protein overexpression has been described in many varieties of human cancers, and its role in MET signaling is essential for cancer stemness and poor prognosis [35]. C-Met has also been shown to activate the WNT/β-catenin signaling cascade to promote stemness and invasion [36]. Our study revealed increased expression and activation of c-Met in cells and tumors lacking MCPIP1 RNase activity. We found that the activation of c-Met is MCPIP1-dependent and is correlated with the acquisition of an undifferentiated/mesenchymal phenotype that is able to induce tumorigenesis. It has been shown that c-Met receptor activation might regulate c-Myc expression together with that of other reprogramming transcription factors, such as Sox2, Klf4, Oct4, and Nanog [37]. Moreover, c-Met signaling can dynamically regulate glioma subpopulations and expand the pool of stem-like cells, and it is correlated with the stem/progenitor phenotype in glioblastoma specimens [37]. Moreover, c-Met is highly expressed in RCC, and c-Met overexpression is a potential poor prognostic marker for RCC patients [38].

c-Met recruits helper molecules to amplify signaling to allow for the activation of the receptor. One of the inducers of c-Met activation and signaling is CD44, which synergizes with the pair CD44/c-Met to potentiate signal transduction from the complex [8]. To explain the increased activation of the c-Met receptor in cells lacking MCPIP1 activity, we found that CD44 expression was significantly increased in cells and tumors lacking MCPIP1 RNase activity, suggesting its dependence on MCPIP1 endonuclease activity. We also observed increased complex formation of CD44 and c-Met in cells expressing MCPIP1 mutations. It seems that the endonuclease activity of MCPIP1, which regulates the expression of CD44, directly influences c-Met activation. Moreover, we found that increased expression of CD44 in ccRCC patient samples was correlated with tumor grade. This finding is consistent with previous research showing that CD44 overexpression or alternative splicing has been described for various types of cancers, including ccRCC [33]. Our observations might explain the increased activation of c-Met, changes in the EMT phenotype and promotion of tumorigenesis.

For the first time, this study reveals an unrecognized role of MCPIP1 in regulating the initiation of tumorigenesis induced by direct and indirect modulation of factors pivotal to tumor initiation. MCPIP1 emerges as a critical regulator of increased activation of the stemness marker c-Met, which is induced by increased CD44 expression and EMT induction.

## Conclusions

This study further explains the molecular mechanism underlying the tumor suppressor function of MCPIP1 and highlights the pathways by which the RNase activity of MCPIP1 controls the activation of oncogenic signaling. Understanding these regulatory mechanisms provides a nuanced perspective on the potential of MCPIP1 as a therapeutic target for interventions aimed at impeding the initiation of cancer. Further studies focused on the effects of MCPIP1 expression on the microenvironment and its relevance to cancer pathogenesis can be derived from this study.

## Materials and methods

### Cell culture

Mouse epithelial kidney cell line TCMK-1 (cat no. CCL-139, Manassas, VA, USA) and Caki-1 (cat no. HTB-46) was obtained from ATCC and was cultured in Eagle’s minimal essential medium (EMEM; Lonza, Basel, Switzerland) supplemented with 10% fetal bovine serum (FBS). The RPTEC/TERT1 (ATCC) cell line (passages 7–17) was cultured in DMEM:F12 medium (ATCC) supplemented with the components of the hTERT RPTEC Growth Kit (ATCC) and G418 at a final concentration of 0.1 mg/ml (Sigma). The HK-2 cells were obtained from ATCC (cat no. CRL-2190) and were cultured in Keratinocyte Serum Free Medium Invitrogen 17,005–042) supplemented with 0.05 mg/ml BPE and 5 ng/ml EGF. HKC-8 cells were kindly provided by the Department of Medical Biotechnology, Jagiellonian University (Krakow, Poland) and cultured in DMEM F12 medium supplemented with 10% fetal bovine serum (FBS) and 1X ITS. The cells in the initial vials were expanded and cryopreserved, and cells were propagated with less than fifteen consecutive passages. All cell lines were routinely tested for mycoplasma by PCR every three months. All cell lines were cultured at 37 °C in a humidified incubator in a 5% CO_2_ atmosphere.

### Stable transduction of MCPIP1 with viral vectors

For stable overexpression of MCPIP1, a doxycycline-dependent lentiviral TetON system was used (pLIX-MCPIP1, pLIX-PURO and the mutant form pLIX D141N). A GFP-expressing lentiviral vector was used. To stably inhibit MCPIP1 in the RPTEC/TERT1 cell line, shRNA (shMCPIP1) and control (shCtrl) lentiviral vectors (Sigma) were used. Briefly, ccRCC cells were plated at 50% confluency in six-well plates. Viral vectors were added at a multiplicity of infection (MOI) of 50 with 6 mg/mL polybrene (Millipore). To increase the transduction efficiency after 24 h, the process was repeated. Cells were incubated with viruses for 24 h, and then the medium was changed. After an additional 24 h, 1 μg/mL puromycin (InvivoGen, San Diego, CA, USA) was added to start selection. To induce MCPIP1 overexpression in the TetON system, 1 μg/mL doxycycline (BioShop) was added for 24–48 h.

### Next generation sequencing

Transcriptome sequencing was performed as described previously [39]. FASTQ raw files are available under Sequence Read Archive (NCBI) accession number PRJNA1131738. Gene Set Enrichment Analysis (GSEA) was performed with GSEA software from Broad Institute v4.3.2. Normalized RNA-seq data was used as an input. Analysis was performed with Hallmark gene set from mouse MSigDB Collections (mh.all.v2023.2.Mm.symbols.gmt). FDR < 0,25 was used to determine significantly enriched gene sets.

### Mouse experiments

Mouse experiments were conducted in accordance with protocols approved by the Institutional Animal Care and Use Committee: II Local Ethics Committee of the Institute of Pharmacology Polish Academy of Sciences (Approval no. 217/2021 and 53/2019). Mice were handled according to the regulations of national and local animal welfare bodies. Six-week-old female NOD-SCID mice (Charles River Laboratory, Wilmington, MA, USA) were kept under specific pathogen-free (SPF) conditions, with water and food provided ad libitum. TCMK-1 GFP shCtrl, shMCPIP1, pLIX PURO or pLIX D141N cells were injected subcutaneously as a cell suspension (5 × 10^6^ cells in PBS with Matrigel 1:1), Caki-1 pLIX PURO or pLIX D141N were injected as a cell suspension 2.5 × 10^6^ cells in PBS. Mice injected with TCMK-1/Caki-1 pLIX-PURO or pLIX-MCPIP1 drank water with doxycycline (200 mg/L) to induce MCPIP1 overexpression. Tumor growth was monitored for 7 weeks. Tumor volume was estimated using caliper measurements, according to the formula: volume = width × depth^2^ of the tumor. After tumor and lung excision, RNA isolation and histologic analysis were performed.

### Patient tissue specimens

Patient tumor tissue was obtained from surgery for renal cancer in the Centre of Oncology, Maria Sklodowska-Curie Memorial Institute (Krakow Branch, Poland). The study was approved by the Hospital Institutional Review Board, and informed consent was obtained from each patient. All human tissue samples were collected using protocols approved by the Local Ethics Committee (Approval no. 68/KBL/OIL/2011). All samples were histologically evaluated by pathologists and diagnosed according to the World Health Organization classification system, specimens were divided into four groups (I–IV) according to histologic grading with the Fuhrman system. A sample of each tumor specimen was frozen in liquid nitrogen and stored at − 80 °C for proteomic analysis or incubated overnight in RNAlater (Invitrogen, Waltham, MA, USA, cat. No. AM7024) at 4 °C and stored at − 80 °C for RNA expression. The analysis of the MCPIP1 and c-Met protein levels and the gene expression microarray included 60 samples, 15 samples from each group (I–IV).

### RNA isolation and quantitative RT-PCR

Total RNA was isolated from cultured cells using the Universal RNA Purification Kit (EURx, Gdańsk, Poland, cat no. E3598-02). RNA was isolated from tumor tissues using fenozol (phenol–chloroform extraction, A&A Biotechnology, Gdańsk, Poland, cat. no. 203–100). The quantity of ribosomal RNA and the presence of DNA contamination were examined using electrophoresis with a 1% denaturing formaldehyde gel. The concentration of total RNA was assessed using a NanoDrop 2000 spectrophotometer (Thermo Fisher Scientific). Reverse transcription was performed using 1 mg of total RNA, oligo(dT) 15 primer (1 μg/μl, Promega, Madison, WI, USA, cat. no. C1101), dNTPs (10 mM, Promega, cat. no. U1330) and M-MLV reverse transcriptase (Promega, cat. no. M1701). Real-time PCR was carried out using SYBRGreen Master Mix (A&A Biotechnology, cat. no. 2008-1000A) and QuantStudio 3 (Applied Biosystems, Waltham, MA, USA). Gene expression was normalized to the expression of elongation factor-2 (EF2; cell lines) or Actb (tissues). The mRNA level in each sample was analyzed in duplicate. The relative levels of transcripts were quantified by the ΔΔCt method. The sequences of primers (Sigma-Aldrich) and annealing temperatures are listed in Supplementary Table S1.

### Western blot analysis

Cultured cells were washed with ice-cold PBS (Lonza), harvested, and lysed with lysis buffer 6 (R&D, cat. no. 895561) for 30 min. The lysates were then centrifuged for 5 min at 15,000 × *g* and 4 °C. SDS-PAGE was conducted with a 10% polyacrylamide gel. After wet transfer to polyvinylidene difluoride membranes (Millipore, cat. no. IPVH00010), the membranes were blocked in 3% BSA in Tris-buffered saline with 0.1% Tween 20 (Sigma-Aldrich). Next, the membranes were incubated with primary antibodies at 4 °C overnight with gentle agitation. On the following day, the membranes were washed three times for 10 min with TBS with 0.1% Tween 20 and incubated with a secondary antibody for 1 h at room temperature (RT) with gentle rocking. Chemiluminescence was detected after a 5-min incubation with Immobilon Western HRP substrate (Millipore, cat no. WBKLS0050) using a ChemiDoc system (Bio-Rad). All antibodies and dilutions are listed in Supplementary Table S2.

### Crystal violet staining of clones

One thousand cells were seeded in six-well plates. After 7 days, cultured cells were washed with PBS and fixed with 100% methanol for 5 min at room temperature. Next, the cells were stained with a 20% crystal violet solution in methanol for 20 min at room temperature. After three washes with PBS, the cells were air dried. All images were acquired using ChemiDoc system (Bio-Rad).

### Flow cytometry of GFP positive cells

Mouse blood were collected at the end of experiment, and next, the erythrocytes were lysed with High-Yield Lyse Fixative-Free Lysing Solution (Life Technologies) for 30 min. Lysed blood samples were diluted 100 × in PBS, and 10 mL of each sample was taken for analysis. Measurement was performed using Attune NxT Acousting Focusing Cytometer and analyzed with Attune software v2.4.

### Proteome-profiler assay

Proteomic analysis was performed using the Proteome Profiler Mouse XL Cytokine Array Kit (R&D Systems, Minneapolis, MN, USA, cat. no. ARY028) according to the manufacturer’s protocol. Mice serum was collected 7 weeks after injection of TCMK-1 cells. 100 μl of each sample was taken per array set. Chemiluminescence was detected after 10 min of incubation with Chemi Reagent Mix (R&D Systems, provided with the kit) using a ChemiDoc system (Bio-Rad, Hercules, CA, USA). The densitometric values of each set of dots were measured using Image Lab 5.2.1 software (Bio-Rad).

### Immunohistochemical (IHC) staining

Fresh tissues were prefixed in pure buffered formaldehyde (Chempur), washed in PBS, incubated for 12 h in 30% sucrose at 4 °C and embedded in OCT (VWR Chemicals). Then, 10-μm slides were cut on a cryostat (Leica) and placed on poly-l-lysine-coated slides. Next, the sections were permeabilized (0.1% Triton X-100 in PBS) and blocked in blocking buffer (5% BSA or 2% powdered milk in PBS) at room temperature for 1 h. Samples were incubated with primary antibodies in 1% BSA in PBS o/n in cold room. On the following day, the slides were washed with PBS and incubated for 1 h at room temperature with secondary antibodies conjugated with Alexa Fluor 594, Alexa Fluor 568, Alexa Fluor 647 or Alexa Fluor 546 (1:1000; Thermo Fisher Scientific) and Hoechst nuclear stain. The slides were mounted with Dako Fluorescent Mounting Medium (Agilent Technologies, cat. no. CS70330-2). Hematoxilin & eosin staining, slides were incubated 10 min in hematoxilin followed by washing in water. Next, slides were incubated 50 s in eosin and washed in water. Samples were incubated 5 min in 95% ethanol, 5 min in 100% ethanol and 5 min in xylene. The slides were mounted with Epredia Consul-Mount (Fisher Scientific, cat. 12,590,017). Picro Sirius Red staining, slides were incubated for 6 min in filtered Picro Sirius Red buffer. Next, washed two times in acidic water and incubated in three different vessels with 100% ethanol for 5 min and xylene for additional 5 min. Samples were mounted with consul-mount. Images were acquired with a Leica DM6 B fluorescence microscope with a × 5, × 10 or × 20 dry objective and Leica LAS X image acquisition software.

### Gene expression microarray analysis

Gene expression analysis was performed using Affymetrix HuGene ST 2.1 microarrays (Affymetrix, Santa Clara, CA, USA) on RNA samples isolated from ccRCC patient tissues as described above. For isolation of total RNA, the EURx Universal RNA Purification Kit (EURx, Gdańsk, Poland, cat no. E3598-02) was used according to the manufacturer’s protocol. The quantity of ribosomal RNA and DNA contamination was examined by electrophoresis with a 1% denaturing formaldehyde gel. The total RNA concentration was measured by a NanoDrop 1000 spectrophotometer (Thermo Fisher Scientific, Waltham, MA, USA). From each sample, 100 ng of total RNA was used to synthesize and amplify ss-cDNA, followed by fragmentation and labeling with biotin. Each step was performed according to the Affymetrix GeneChip WT PLUS Reagent Kit Manual. Next, 10 μg cRNA was hybridized for 16 h at 48 °C on the Affymetrix HuGene2.1 ST Array Strip and then washed and stained in the Affymetrix Gene Atlas Fluidics Station. Each array strip was scanned using the GeneAtlas Imaging Station. The data were normalized with Expression Console Software 1.4.1 with the RMA algorithm and analyzed using Affymetrix Transcriptome Analysis Console (TAC) Software 3.1 with one-way ANOVA between subjects (unpaired). Next, we followed the Minimum Information About a Microarray Gene Experiment (MIAME) guidelines and deposited raw and processed data in the Gene Expression Omnibus (GEO) repository under accession number GSE150404.

### Coimmunoprecipitation

To determine the interaction between CD44 and Met protein, co-immunoprecipitation with anti-Met (D1C2) XP® Rabbit mAb #8198 (Cell Signaling) was performed. 1.5 × 10^6 Caki-1 pLIX PURO and pLIX D141N cells were seeded on two 150 mm cell culture dishes. The day after seeding, the cells were stimulated with doxycycline. After 48 h of stimulation with doxycycline, the cells were washed with ice-cold PBS (Lonza), harvested, and lysed with M-PER™ buffer (Thermo Scientific™), with protease and phosphatase inhibitors. The lysates were then centrifuged for 5 min at 15,000 × g, 4 °C, and transferred to new tubes. A BCA assay was performed to determine protein concentration in the cell lysates. For co-immunoprecipitation, 500 µg of protein per sample was used, both for the sample with antibodies and the control sample. HNTG buffer with protease inhibitors was added to bring the final sample volume to 500 µl. Samples were precleaned by 2-h incubation at 4 °C in a tube rotator with 35 µl of Pierce™ Protein G Agarose beads (Thermo Scientific™). Agarose beads were prepared by washing with 400 µl of HNTG buffer with protease inhibitors. After preclearing, samples were centrifuged for 4 min at 4000 rpm at 4 °C. Supernatants were transferred to new tubes. Samples were incubated overnight with 10 µl of Met (D1C2) antibody in a tube rotator at 4 °C. Nothing was added to the control samples. Agarose beads blocked by 1-h incubation in 1% BSA in PBS and washed three times with ice-cold PBS and once with HNTG with protease inhibitors were added to the samples (30 µl per sample). After 1.5 h of incubation in a tube rotator at 4 °C, beads were centrifuged for 3 min at 3000 rpm at 4 °C and washed two times by adding 1.5 ml of HNTG buffer with 1% BSA and centrifugation for 4 min at 2000 rpm at 4 °C. After the last centrifugation, beads were suspended in 25 µl of Laemmli buffer with β-mercaptoethanol and incubated for 10 min at 95 °C. Samples were centrifuged for 5 min at 2000 rpm, and at maximum speed for one second. The supernatant was used in western blot analysis.

### Transient transfection with small interfering RNA (siRNA)

CD44 inhibition was examined using siCD44 (Thermo Fisher Scientific, cat no. AM16708) with control (Thermo Fisher Scientific, cat. No. AM4611). Shortly, cells were seeded one day prior transfection into 12-well plates in approximately 60% of confluency. Transfection was performed using the JetPrime transfection reagent (Polyplus Transfection) according to the protocol provided by the manufacturer. Caki-1 pLIX PURO and pLIX D141N cells were also stimulated with doxycyclin. 48 h after transfection, cell lysates were prepared and used for further analysis.

### Statistical analysis

All in vitro experiments were conducted at least three times independently. The number of animals is indicated in the figure legends. All results are shown as the mean ± SD. For graph preparation and statistical analysis, GraphPad Prism 7 (San Diego, CA, USA) was used. For comparison of two groups, the Student’s *t*-test or Mann–Whitney test were used. *P* values are marked with asterisks in graphs (*P < 0.05; **P < 0.01; ***P < 0.001; ****P < 0.0001 versus the control).

## Supplementary Information

Supplementary Material 1.

Supplementary Material 2.

Supplementary Material 3.

## Data Availability

For microarray analysis we followed the Minimum Information About a Microarray Gene Experiment (MIAME) guidelines and deposited raw and processed data in the Gene Expression Omnibus (GEO) repository under accession number GSE150404. For transcriptome sequencing FASTQ raw files are available under Sequence Read Archive (NCBI) accession number PRJNA1131738. Supplementary information containing additional data supporting our findings is also provided.

## Abbreviations

CICs: Cancer-initiating cells
CSCs: Cancer stem cells
EMT: Epithelial-to-mesenchymal transition
FN1: Fibronectin
HIF1: Hypoxia inducible factor 1
IL-6: Interleukin-6
IL-1β: Interleukin-1β
MAPK: Mitogen-activated protein kinase
MCPIP1: Monocyte chemoattractant protein-1-induced protein 1
NF-Κb: Nuclear factor-kappa B
PIN: PilT N-terminus-like
PECAM-1, CD31: Platelet endothelial cell adhesion molecule
RPTEC / TERT1: Renal proximal tubule epithelial cells TERT-immortalized 1
TIMP3: Tissue inhibitor of metalloproteinases
TCMK1: Transformed C3H mouse kidney-1 cells
TAMs: Tumor-associated macrophages
TNF-α: Tumor necrosis factor-alpha
VEGF: Vascular endothelial growth factor
VIM: Vimentin
