# Supplementary material for: The endonuclease activity of MCPIP1 controls the neoplastic transformation of epithelial cells via the c-Met/CD44 axis
Source: Cell Commun Signal. 2025 Jan 15;23:28. doi: 10.1186/s12964-025-02029-x (PMC11734405; doi:10.1186/s12964-025-02029-x)
Supplement: Supplementary file 1 — Supplementary Material 1. [file 12964_2025_2029_MOESM1_ESM.pdf]

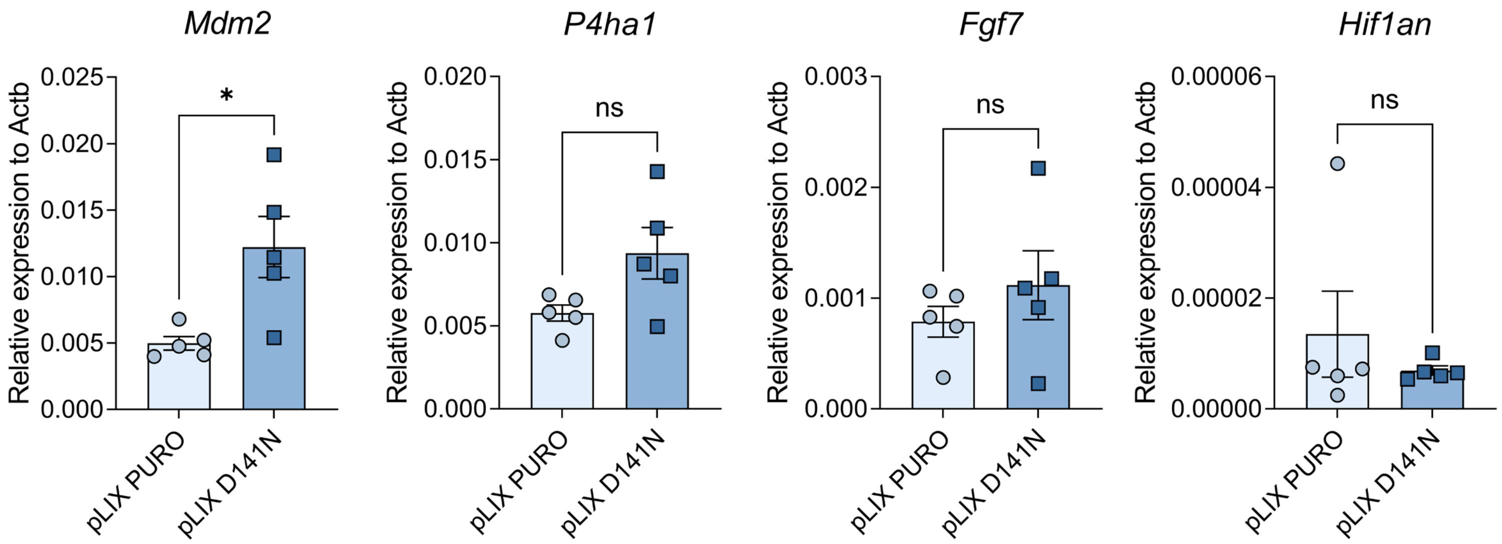

Mdm2
P4ha1
Fgf7
Hif1an
Relative expression to Actb
0.025
0.020
0.015
0.010
0.005
0.000
0.003
0.002
0.001
0.00006
0.00004
0.00002
0.00000
*
ns
pLIX PURO
pLIX D141N

Supplementary Fig. 1 Validation of transcripts selected on the basis of NGS. Relative expression of transcripts selected from Fig. 1E Heat map: *Mdm2*, *P4ha1*, *Fgf7*, *Hif1an* in tumor tissues. Animal studies involved 10 NOD-SCID mice: TCMK-1 pLIX PURO, N = 5; TCMK-1 pLIX D141N, N = 5. The results are presented as the mean ± SD. P values were estimated using Student *t*-test, except *Hif1an* where was used Mann-Whitney test. *P<0.05.

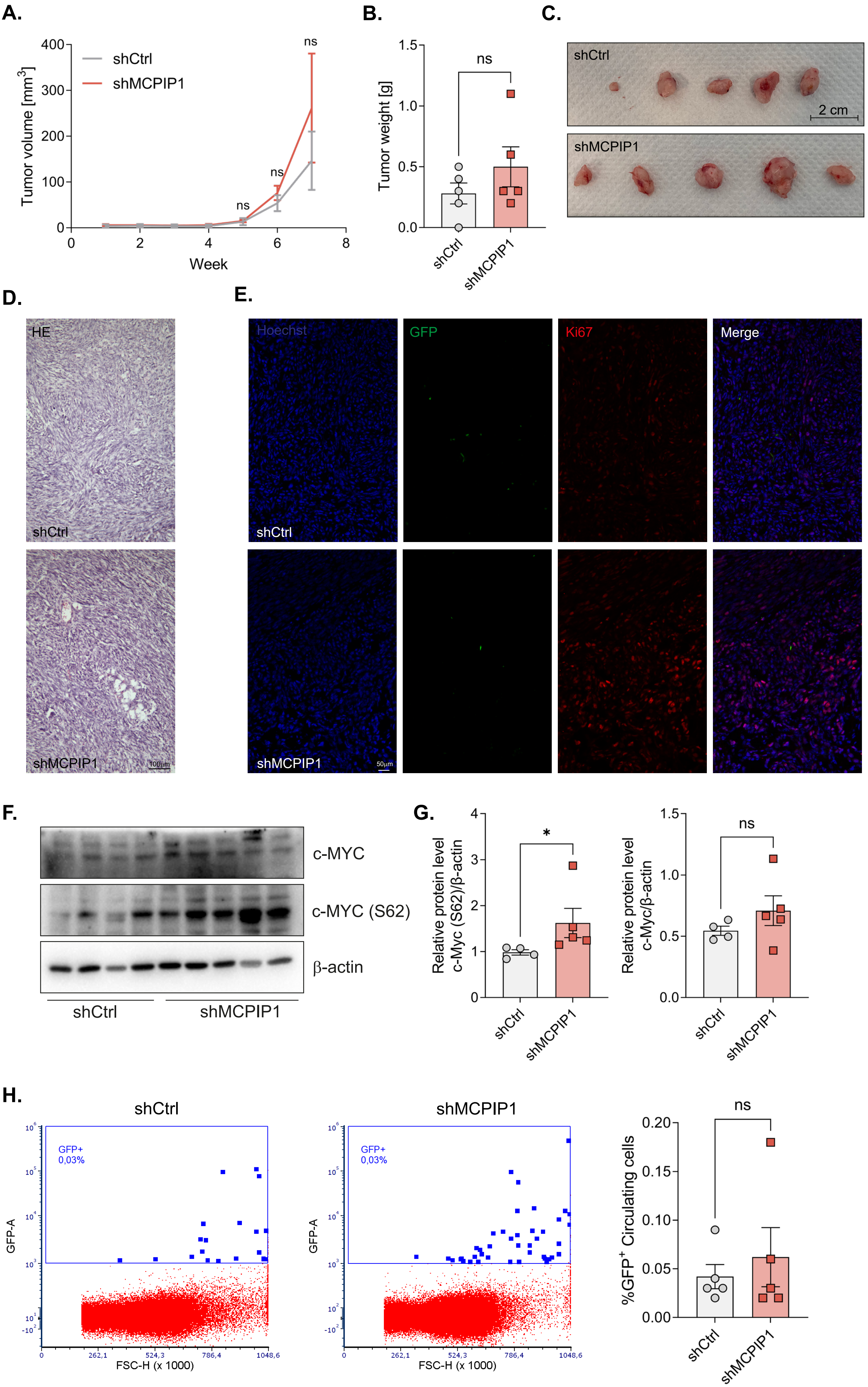
A.
Tumor volume [mm3]
shCtrl
shMCPIP1
ns
Week
B.
Tumor weight [g]
ns
shCtrl
shMCPIP1
C.
shCtrl
2 cm
shMCPIP1
D.
HE
shCtrl
shMCPIP1
E.
Hoechst
GFP
Ki67
Merge
shCtrl
shMCPIP1
F.
c-MYC
c-MYC (S62)
β-actin
shCtrl
shMCPIP1
G.
Relative protein level c-Myc (S62)/β-actin
*
Relative protein level c-Myc/β-actin
ns
H.
shCtrl
shMCPIP1
GFP+ 0,03%
GFP-A
FSC-H (x 1000)
%GFP+ Circulating cells
ns

Supplementary Fig. 2 Effect of MCPIP1 downregulation on tumor growth *in vivo*. A. Caliper measurements of tumor volume during 7 weeks. **B.** Weight of tumors. **C.** Representative images of extracted tumors. **D.** Representative images of tumor sections after hematoxylin and eosin staining. **E.** Ki67 immunofluorescent staining of OCT tumor sections, representative images with Hoechst used to visualize nuclei. TCMK-1 cells were GFP positive. **F.** Western blot result of tumors fragments with b-actin as a loading control. **G.** Densitometric analysis of Western blot result. **H.** Flow cytometer analysis of circulating GFP-positive TCMK-1 cells in mouse lysed blood. Tumors and blood were collected 7 weeks after subcutaneous injection of cells. Animal studies involved 10 NOD-SCID mice: TCMK-1 shCtrl, N = 5; TCMK-1 shMCPIP1, N = 5; except Western blot where shCtrl N=4. The results are presented as the mean ± SD. P values were estimated using Student *t*-test or Mann-Whitney test (c-MYC S62, GFP+ circulating cells). *P<0.05.

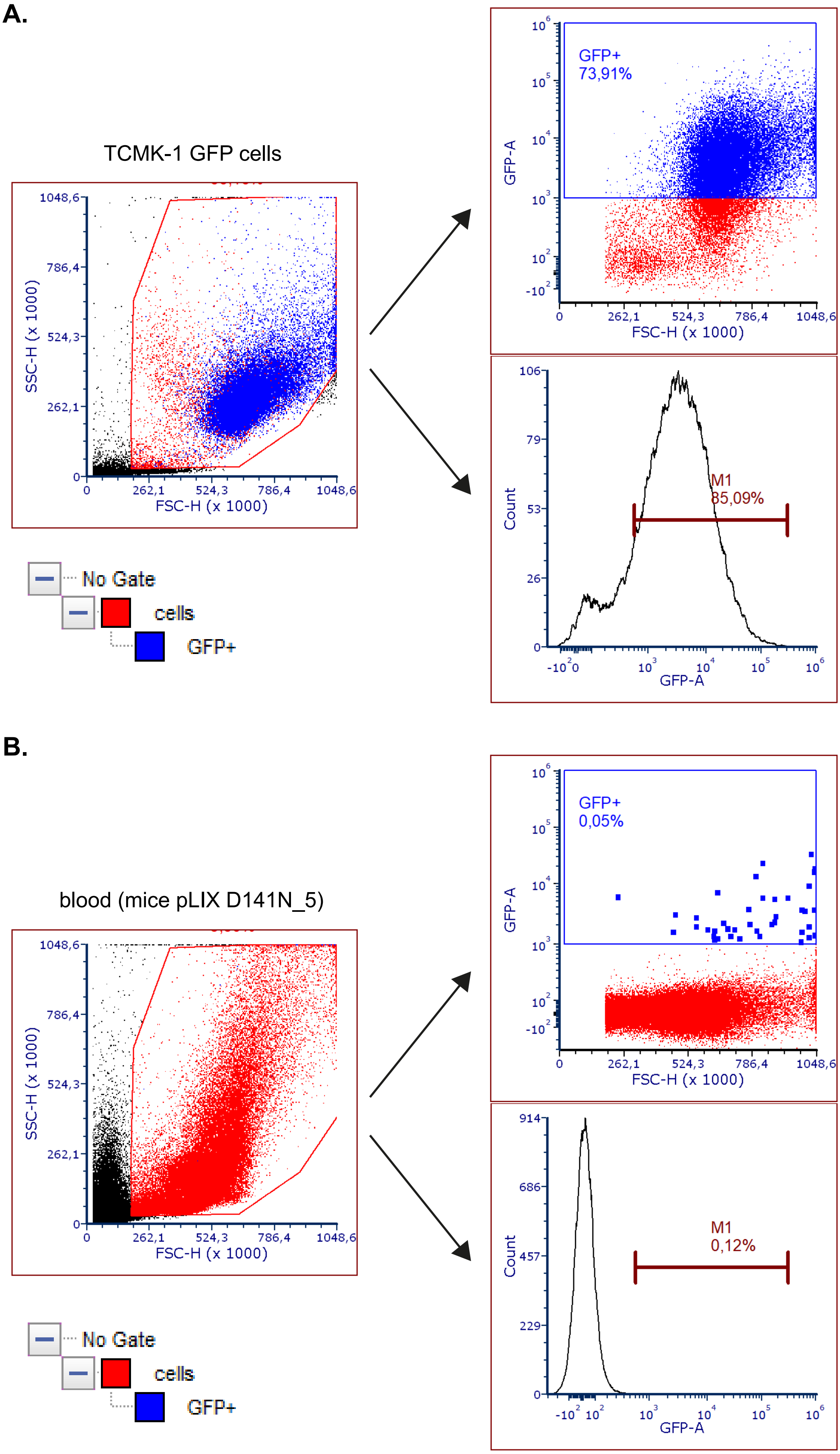
A.
TCMK-1 GFP cells
SSC-H (x 1000)
FSC-H (x 1000)
No Gate
cells
GFP+
GFP+
73,91%
GFP-A
Count
M1
85,09%
B.
blood (mice pLIX D141N_5)
SSC-H (x 1000)
FSC-H (x 1000)
No Gate
cells
GFP+
GFP+
0,05%
GFP-A
Count
M1
0,12%

Supplementary Fig. 3 Gating strategy of GFP positive cells in the blood. A. Gating strategy of TCMK-1 GFP cells. Cells marked in blue are GFP positive. **B.** Gating strategy of mice blood 7 weeks after subcutaneous injection of TCMK-1 GFP positive cells.

A.

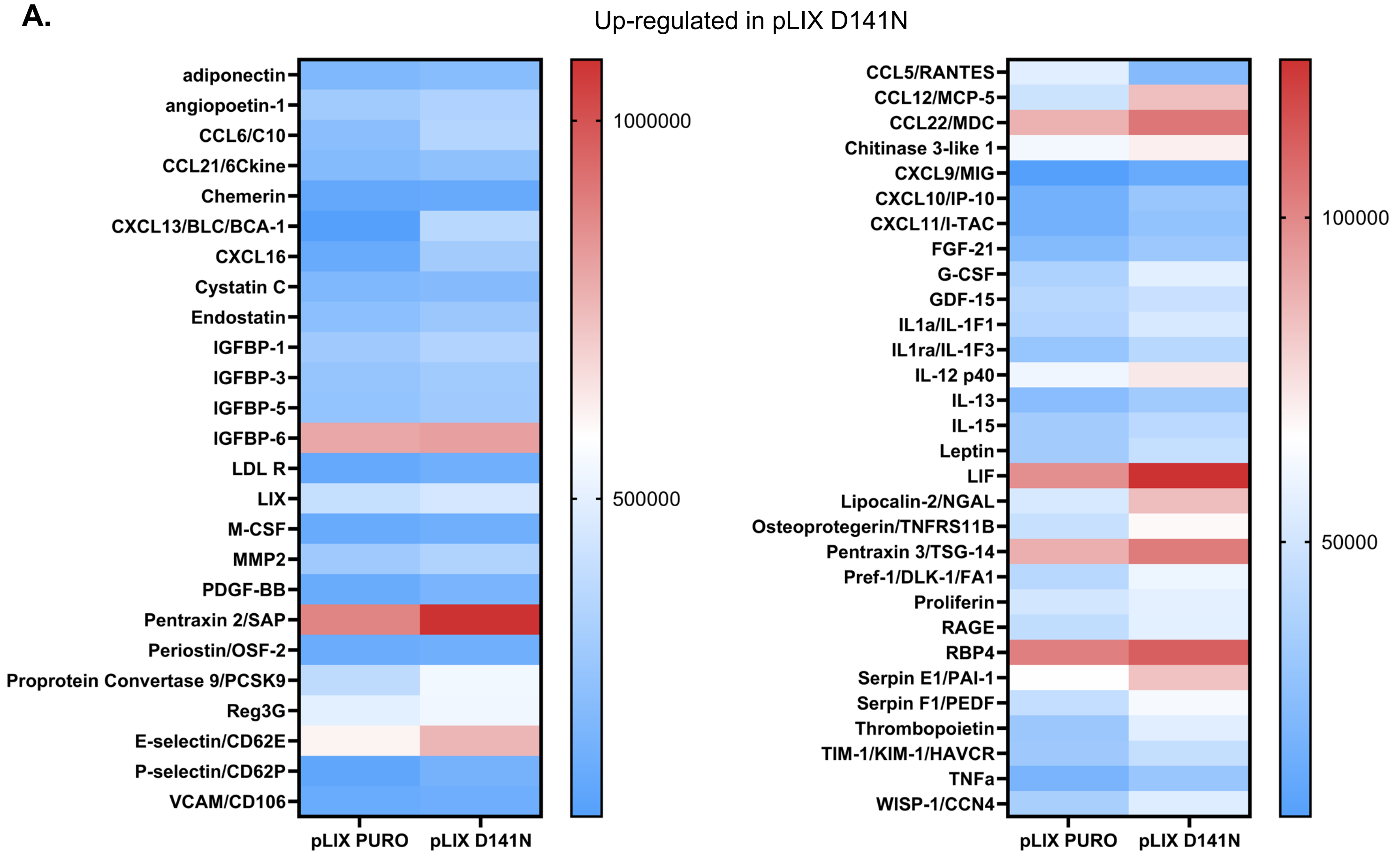

Up-regulated in pLIX D141N
adiponectin
angiopoetin-1
CCL6/C10
CCL21/6Ckine
Chemerin
CXCL13/BLC/BCA-1
CXCL16
Cystatin C
Endostatin
IGFBP-1
IGFBP-3
IGFBP-5
IGFBP-6
LDL R
LIX
M-CSF
MMP2
PDGF-BB
Pentraxin 2/SAP
Periostin/OSF-2
Proprotein Convertase 9/PCSK9
Reg3G
E-selectin/CD62E
P-selectin/CD62P
VCAM/CD106
pLIX PURO
pLIX D141N
1000000
500000
CCL5/RANTES
CCL12/MCP-5
CCL22/MDC
Chitinase 3-like 1
CXCL9/MIG
CXCL10/IP-10
CXCL11/I-TAC
FGF-21
G-CSF
GDF-15
IL1a/IL-1F1
IL1ra/IL-1F3
IL-12 p40
IL-13
IL-15
Leptin
LIF
Lipocalin-2/NGAL
Osteoprotegerin/TNFRS11B
Pentraxin 3/TSG-14
Pref-1/DLK-1/FA1
Proliferin
RAGE
RBP4
Serpin E1/PAI-1
Serpin F1/PEDF
Thrombopoietin
TIM-1/KIM-1/HAVCR
TNFa
WISP-1/CCN4
pLIX PURO
pLIX D141N
100000
50000

B.

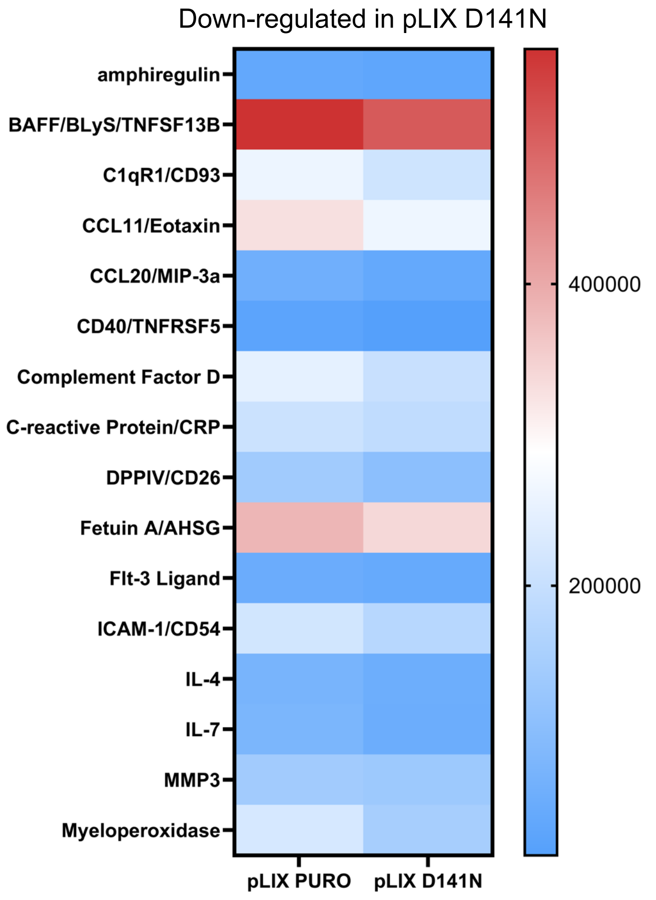

Down-regulated in pLIX D141N
amphiregulin
BAFF/BLyS/TNFSF13B
C1qR1/CD93
CCL11/Eotaxin
CCL20/MIP-3a
CD40/TNFRSF5
Complement Factor D
C-reactive Protein/CRP
DPPIV/CD26
Fetuin A/AHSG
Flt-3 Ligand
ICAM-1/CD54
IL-4
IL-7
MMP3
Myeloperoxidase
pLIX PURO
pLIX D141N
400000
200000

Supplementary Fig. 4 Proteome profiler densitometric analysis. A. Heat maps with all proteins up-regulated in serum of mice from group pLIX D141N. **B.** Heat map with all proteins down-regulated in serum of mice from group pLIX D141N. Each column represents mean of two serum samples. This study involved 4 NOD-SCID mice: TCMK-1 pLIX PURO, N=2; TCMK-1 pLIX D141N, N=2.

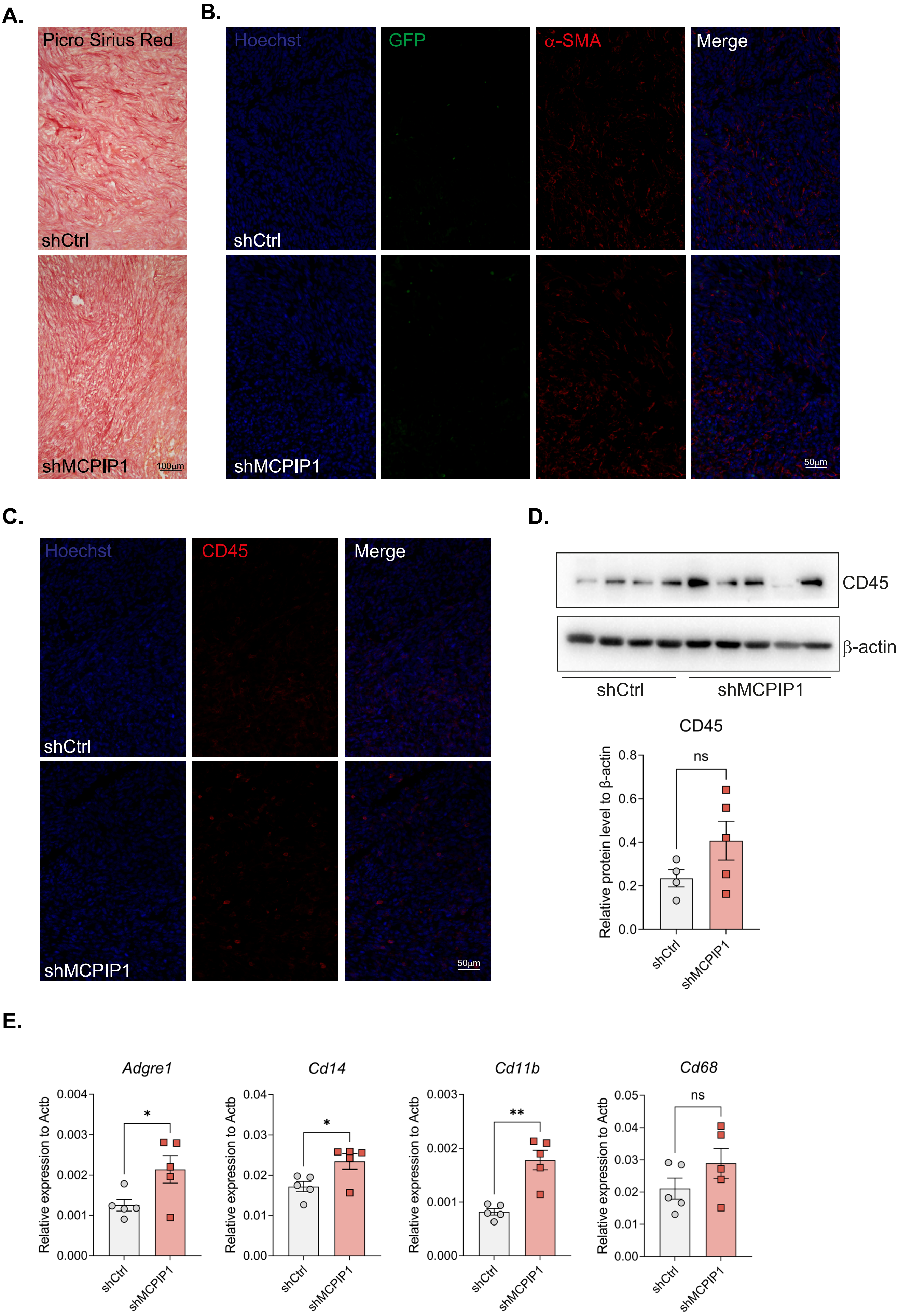
A.
Picro Sirius Red
shCtrl
shMCPIP1
100μm
B.
Hoechst
GFP
α-SMA
Merge
shCtrl
shMCPIP1
50μm
C.
Hoechst
CD45
Merge
shCtrl
shMCPIP1
50μm
D.
CD45
β-actin
shCtrl
shMCPIP1
CD45
ns
Relative protein level to β-actin
0.0
0.2
0.4
0.6
0.8
shCtrl
shMCPIP1
E.
Adgre1
Cd14
Cd11b
Cd68
Relative expression to Actb
*
*
**
ns
0.000
0.001
0.002
0.003
0.004
0.00
0.01
0.02
0.03
0.04
0.05
shCtrl
shMCPIP1

Supplementary Fig. 5 Effect of MCPIP1 downregulation on local microenvironment. A. Representative images of tumor sections stained with Picrosirius Red. **B.** a-SMA immunofluorescent staining of OCT tumor sections, representative images with Hoechst used to visualize nuclei. TCMK-1 cells were GFP positive. **C.** CD45 immunofluorescent staining of OCT tumor sections, representative images with Hoechst used to visualize nuclei. **D.** Western blot result of tumors fragments with b-actin as a loading control. On the bottom densitometric analysis. **E.** Relative expression of immune response factors such as *Adgre1*, *Cd14*, *Cd11b* and *Cd68* in tumor tissue. Animal studies involved 10 NOD-SCID mice: TCMK-1 shCtrl, N = 5; TCMK-1 pLIX shMCPIP1, N = 5; except Western blot where shCtrl N=4. The results are presented as the mean ± SD. P values were estimated using Student *t*-test or Mann-Whitney test (*Cd206*, *Cd14*). *P<0.05; **P<0.01.

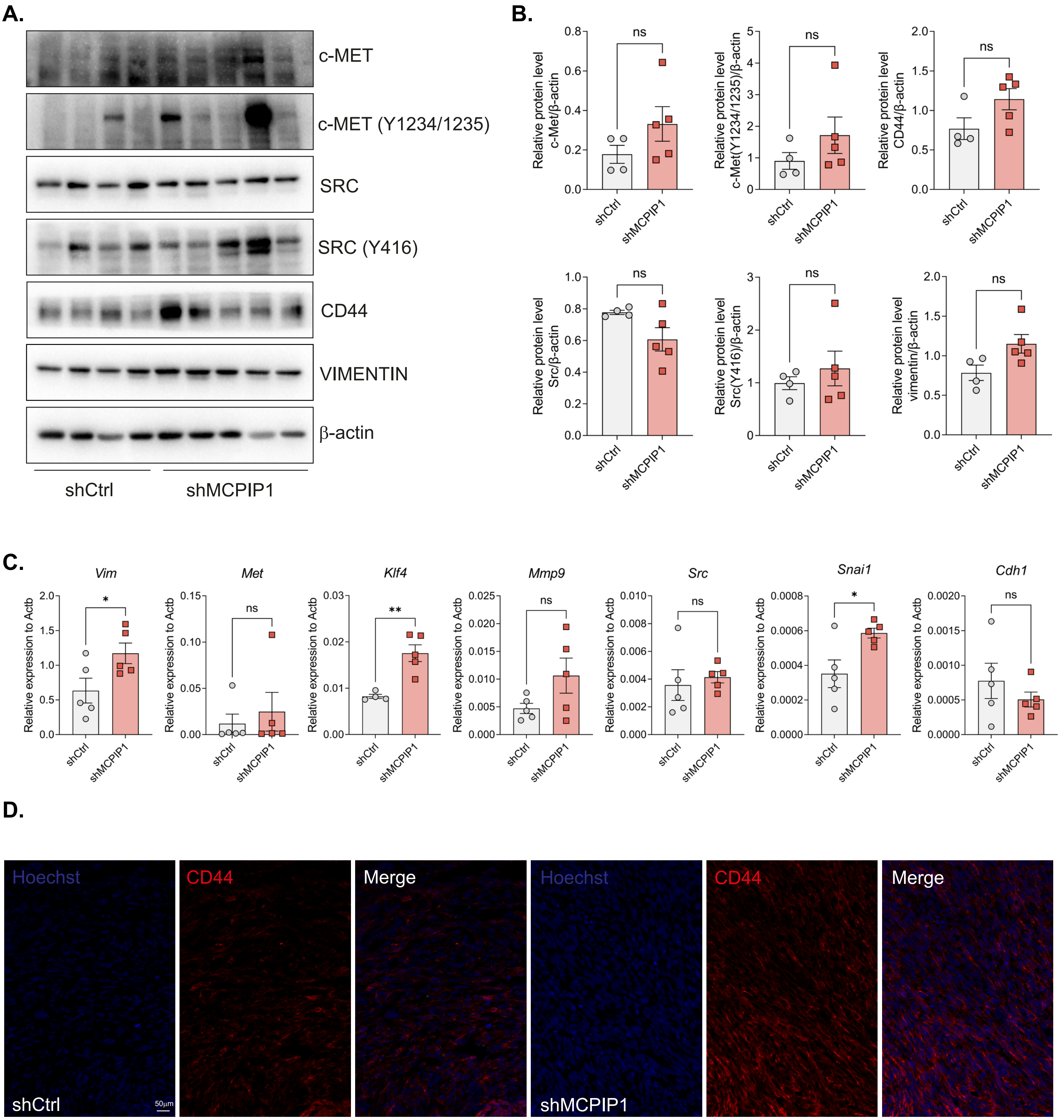

A.
c-MET
c-MET (Y1234/1235)
SRC
SRC (Y416)
CD44
VIMENTIN
β-actin
shCtrl
shMCPIP1
B.
Relative protein level c-Met/β-actin
Relative protein level c-Met(Y1234/1235)/β-actin
Relative protein level CD44/β-actin
Relative protein level Src/β-actin
Relative protein level Src(Y416)/β-actin
Relative protein level vimentin/β-actin
ns
C.
Vim
Met
Klf4
Mmp9
Src
Snai1
Cdh1
Relative expression to Actb
*
**
ns
D.
Hoechst
CD44
Merge
shCtrl
shMCPIP1
50μm

Supplementary Fig. 6 Effect of MCPIP1 downregulation on changes in factors involved in tumor cell stemness and EMT. A. Western blot result of tumors fragments with b-actin as a loading control. **B.** Densitometric analysis of western blot results. **C.** Relative expression of factors involved in tumor growth, progression and EMT *Vim*, *Met*, *Klf4*, *Mmp9*, *Src*, *Snai1* and *Cdh1* in tumor tissues. **D.** CD44 immunofluorescent staining of OCT tumor sections, representative images with Hoechst used to visualize nuclei. Animal studies involved 10 NOD-SCID mice: TCMK-1 shCtrl, N = 5; TCMK-1 shMCPIP1, N = 5; except Western blot where shCtrl N=4. The results are presented as the mean ± SD. P values were estimated using Student *t*-test or Mann-Whitney test (c-MET Y1234/1235, *Met*). *P<0.05; **P<0.01.

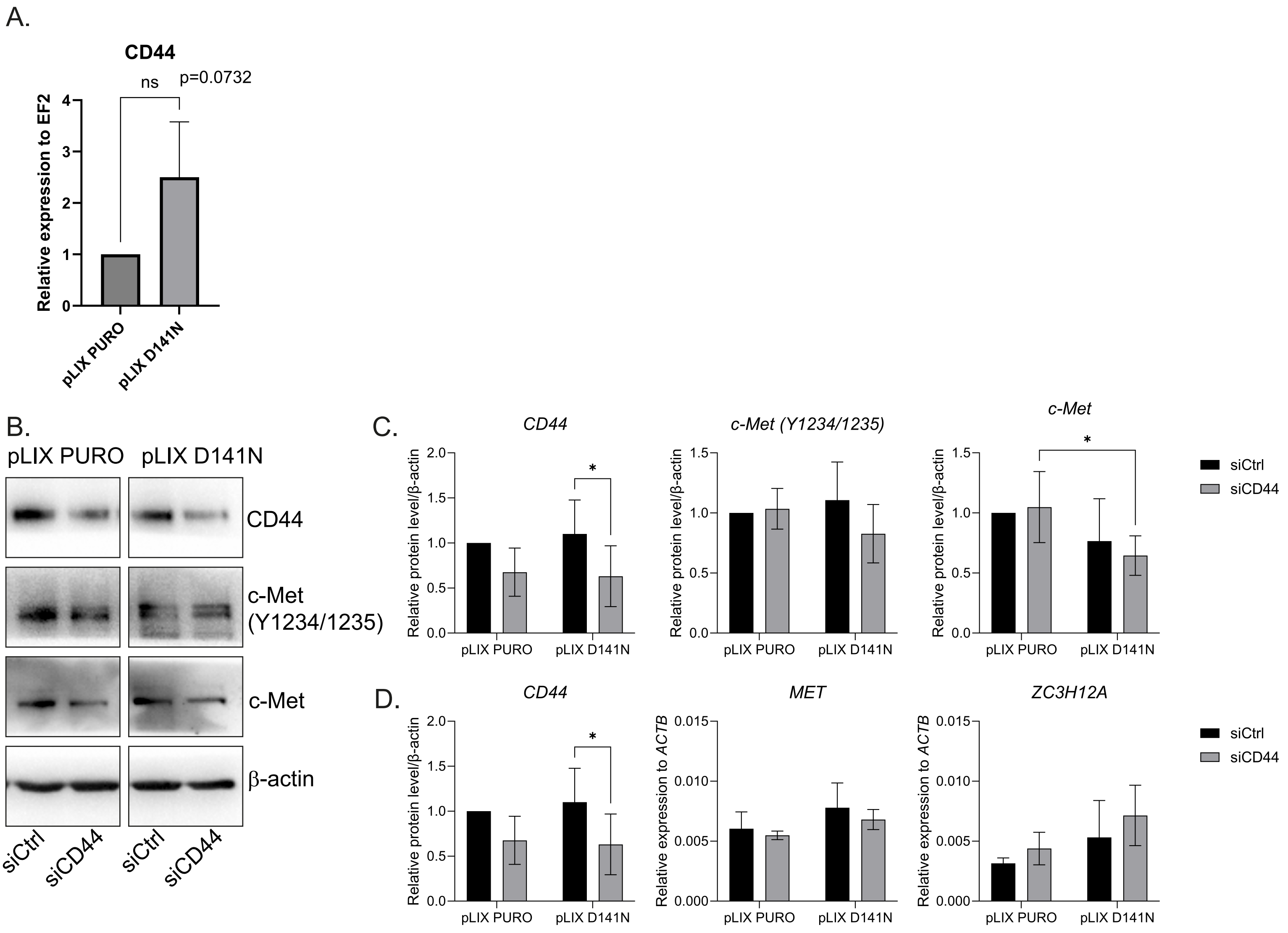
A.
CD44
ns
p=0.0732
Relative expression to EF2
0
1
2
3
4
pLIX PURO
pLIX D141N
B.
pLIX PURO
pLIX D141N
CD44
c-Met (Y1234/1235)
c-Met
β-actin
siCtrl
siCD44
siCtrl
siCD44
C.
CD44
c-Met (Y1234/1235)
c-Met
Relative protein level/β-actin
0.0
0.5
1.0
1.5
2.0
*
pLIX PURO
pLIX D141N
siCtrl
siCD44
D.
CD44
MET
ZC3H12A
Relative protein level/β-actin
Relative expression to ACTB
0.000
0.005
0.010
0.015
*
pLIX PURO
pLIX D141N
siCtrl
siCD44

Supplementary Fig. 7 Effect of MCPIP1 mutation and siCD44 on the level of c-Met. A. Expression of *ZC3H12A* in Caki-1 cells. **B.** Representative western blot of Caki-1 cells (pLIX PURO or pLIX D141N) after transfection with control siRNA or siCD44 with b-actin as a loading control. **C.** Densitometric analysis of western blots results. **D.** Relative expression of transcripts after transfection with siCD44. The results are presented as the mean ± SD. P values were estimated using two-way ANOVA, *P<0.05.
