# Supplementary material for: The endonuclease activity of MCPIP1 controls the neoplastic transformation of epithelial cells via the c-Met/CD44 axis
Source: Cell Commun Signal. 2025 Jan 15;23:28. doi: 10.1186/s12964-025-02029-x (PMC11734405; doi:10.1186/s12964-025-02029-x)
Supplement: Supplementary file 2 — Supplementary Material 2. [file 12964_2025_2029_MOESM2_ESM.docx]

| Gene | Primer sequence | Annealing temperature |
| --- | --- | --- |
| ZC3H12A | F 5’ GGAAGCAGCCGTGTCCCTATG 3’  R 5’ TCCAGGCTGCACTGCTCACTC 3’ | 62 °C |
| MET | F 5’ CATCTCAGAACGGTTCATGCC 3’  R 5’ TGCACAATCAGGCTACTGGG 3’ | 62 °C |
| MMP9 | F 5’ CGCAGACATCGTCATCCAGT 3’  R 5’ GGATTGGCCTTGGAAGATGA 3’ | 62 °C |
| Twist | F 5’ GTCCGCAGTCTTACGAGGAG 3’  R 5’ TGGAGGACCTGGTAGAGGAA 3’ | 62 °C |
| VIM | F 5’ TCTACGAGGAGGAGATGCGG 3’  R 5’ GGTCAAGACGTGCCAGAGAC 3’ | 62 °C |
| SNAI2 | F 5’ TGTTGCAGTGAGGGCAAGAA 3’  R 5’ GACCCTGGTTGCTTCAAGGA 3’ | 62 °C |
| OCT4 | F 5’ CCTTCGCAAGCCCTCATTTCA 3’  R 5’ CCCACAGAACTCATACGGCG 3’ | 60 °C |
| IL6 | F 5’ GCAGAAAAAGGCAAAGAATC 3’  R 5’ CTACATTTGCCGAAGAGC 3’ | 62 °C |
| CD44 | F 5’ TTATCAGGAGACCAAGACAC 3’  R 5’ ATCAGCCATTCTGGAATTTG 3’ | 60 °C |
| PROM1 | F 5’ AAGCATTGGCATCTTCTATG 3’  R 5’ TTTGCTCTGGAGTTTCATTC 3’ | 60 °C |
| ACTB | F 5’ CAAGAGATGGCCACGGCTGCTT 3’  R 5’ CAGGTCTTTGCGGATGTCCACG 3’ | 60 °C |
| FN1 | F 5’ CAGTGGGAGACCTCGAGAAG 3’  R 5’ TCCCTCGGAACATCAGAAAC 3’ | 60 °C |
| Il6 | F 5’ GACAAAGCCAGAGTCCTTCAGAG 3’  R 5’ TTGGATGGTCTTGGTCCTTAGCC 3’ | 62 °C |
| Met | F 5’ CGACAAATACGTTGAAATGC 3’  R 5’GATCTACATAGGAGAATGCAC 3’ | 60 °C |
| Vim | F 5’ GAACCTGAGAGAAACTAACC 3’  R 5’ GATGCTGAGAAGTCTCATTG 3’ | 62 °C |
| Snai2 | F 5’ GACACATTAGAACTCACACTG 3’  R 5’ GACATTCTGGAGAAGGTTTTG 3’ | 60 °C |
| Zeb2 | F 5’ GACACATTAGAACTCACACTG 3’  R 5’ GACATTCTGGAGAAGGTTTTG 3’ | 60 °C |
| Twist | F 5’ GAGACCTAGATGTCATTGTTTC 3’  R 5’ GAATTTGGTCTCTGCTCTTC 3’ | 60 °C |
| Snai1 | F 5’ AGTTGACTACCGACCTTG 3’  R 5’ AAGGTGAACTCCACACAC 3’ | 60 °C |
| Src | F 5’ AATAACACAGAGGGAGACTG 3’  R 5’ATTCCCGTCTAGTGATCTTG 3’ | 60 °C |
| Myc | F 5’ TTTTGTCTATTTGGGGACAG 3’  R 5’ CATAGTTCCTGTTGGTGAAG 3’ | 58 °C |
| Cd206 | F 5’ CGTGGATTCCTTTCTATGGC 3’  R 5’ ACACAATCATTCCGTTCACCA 3’ | 58 °C |
| Dpp4 | F 5’ CTTCTATTCTGATGAGTCACTG 3’  R 5’ CATAAATAGTGATCCCCTCTTG 3’ | 62 °C |
| Adgre1 | F 5’ TTTCAAATGGATCCAGAAGG 3’  R 5’ CAGAAGGAAGCATAACCAAG 3’ | 60 °C |
| Cd14 | F 5’ CTCTGTCCTTAAAGCGGCTTAC 3’  R 5’ GTTGCGGAGGTTCAAGATGTT 3’ | 58 °C |
| Cd11b | F 5’ AAGATCTTTGCAATTGAGGG 3’  R 5’CTCTGGTTGTGTTGATGAAG 3’ | 60 °C |
| Cd68 | F 5’ TATAGCCCAAGGAACAGAGG 3’  R 5’ TATGAGTGACAGTTGTGGGT 3’ | 58 °C |
| Cd44 | F 5’ GTACTATTAGGAGTTGTGCTTG 3’  R 5’ GATATAGACAGAATCAGCACC 3’ | 62 °C |
| Cdh1 | F 5’ CATGTTCACTGTCAATAGGG 3’  R 5’ GTGTATGTAGGGTAACTCTCTC 3’ | 58 °C |
| Klf4 | F 5’ CCCCTCTCTCCATTATCAAG 3’  R 5’ CTCTTGGTATAGGTTTTGCC 3’ | 60 °C |
| Mmp9 | F 5’ CTCTGCTGCCCCTTACCAG 3’  R 5’ AGCGGTACAAGTATGCCTCTGC 3’ | 62 °C |
| P4ha1 | F 5’ GAATTAGCAAAAGTGCTTGG 3’  R 5’ TCATCTTTCCTTGCAAAGCT 3’ | 58 °C |
| Fgf1 | F 5’ ACAGAACAAAAGTCAAGGAG 3’  R 5’ TGATGAGAGAAAAAGTCGTC 3’ | 58 °C |
| Hif1an | F 5’ AACATTGAGAAGATGCTTGG 3’  R 5’ ATACTAGTGCTTGGAGTGTC 3’ | 58 °C |
| Actb | F 5’ CACTGTCGAGTCGCGTCCA 3’  R 5’ TGACCCATTCCCACCATCAC 3’ | 60 °C |

Supplementary Table S1. List of primer’s sequences.
