# Supplementary material for: The endonuclease activity of MCPIP1 controls the neoplastic transformation of epithelial cells via the c-Met/CD44 axis
Source: Cell Commun Signal. 2025 Jan 15;23:28. doi: 10.1186/s12964-025-02029-x (PMC11734405; doi:10.1186/s12964-025-02029-x)
Supplement: Supplementary file 3 — Supplementary Material 3. [file 12964_2025_2029_MOESM3_ESM.docx]

| Antibody | Dilution | Producer (cat no.) |
| --- | --- | --- |
| **Rabbit anti-GAPDH** | 1:1000; 3 % BSA in TBST | Cell Signaling (5174) |
| **Mouse anti-b-actin** | 1:1000; 3 % BSA in TBST | Sigma (1978) |
| **Rabbit anti-MCPIP1** | 1:1000; 3 % BSA in TBST | GeneTex (gtx110807) |
| **Rabbit anti-Src** | 1:1000; 3 % BSA in TBST | Cell Signaling (2123T) |
| **Rabbit anti-Phospho Src (Y416)** | 1:1000; 3 % BSA in TBST | Cell Signaling (2101S) |
| **Mouse anti-c-Met** | 1:1000; 3 % BSA in TBST | Cell Signaling (3127S) |
| **Rabbit anti-Phospho c-Met (Y1234/1235)** | 1:1000; 3 % BSA in TBST | Cell Signaling (3077S) |
| **Rabbit anti-CD44** | 1:1000; 3 % BSA in TBST | Abcam (157107) |
| **Rabbit anti c-Myc** | 1:1000; 3 % BSA in TBST | Cell Signaling (9402S) |
| **Rabbit anti c-Myc (S62)** | 1:1000; 3 % BSA in TBST | Cell Signaling (13748S) |
| **Rabbit anti- a-SMA** | 1:1000; 3 % BSA in TBST | Cell Signaling (19245S) |
| **Rabbit anti-CD45** | 1:1000; 3 % BSA in TBST | Cell Signaling (70257) |
| **Rabbit anti-CD133** | 1:1000; 3 % BSA in TBST | Cell Signaling (48082) |
| **Rabbit anti-vimentin** | 1:1000; 3 % BSA in TBST | Cell Signaling (5741S) |
| **Rat anti-CD31** | 1:50; 1 % BSA in PBS with 0.03% Triton x100 | BD Pharmingen (550274) |
| **Rabbit anti-CD68** | 1:50; 1 % BSA in PBS with 0.03% Triton x100 | Abcam (ab125212) |
| **Rat anti-CD206** | 1:50; 1 % BSA in PBS with 0.03% Triton x100 | Bio-Rad (MCA2235GA) |
| **Goat anti-rabbit IgG-HRP** | 1:4000; 3 % BSA in TBST | Santa Cruz  Biotechnology |
| **Goat anti-mouse IgG-HRP** | 1:4000; 3 % BSA in TBST | Santa Cruz  Biotechnology |

Supplementary Table S2. List of antibodies with dilutions.
